# Efficacy of glutamine supplementation in postoperative patients undergoing hepatic surgery: a systematic review and meta-analysis of randomized controlled trials

**DOI:** 10.1016/j.clinsp.2026.101086

**Published:** 2026-09-01

**Authors:** Xuezheng Meng, Mingming Zhang, Xinran Wang

**Affiliations:** General Surgery Department, Xuanwu Hospital, Capital Medical University, Beijing, China

**Keywords:** Glutamine, Hepatectomy, Liver transplantation, Systematic review, Meta-analysis

## Abstract

**Background:**

The efficacy and safety of glutamine supplementation in patients receiving hepatic surgery remain controversial.

**Methods:**

A search strategy incorporating the terms “glutamine”, “liver transplantation”, and “hepatectomy” was applied across eight literature databases. Study quality was evaluated via the Risk of Bias 2.0 (ROB 2.0) tool, while statistical analyses were undertaken through R 4.4.1.

**Results:**

18 studies involving 1198 patients were encompassed. Meta-analysis revealed that immune factors Immunoglobulin A (IgA), Immunoglobulin M (IgM), Cluster of Differentiation 3 (CD3), Cluster of Differentiation 4 (CD4), and CD4/Cluster of Differentiation 8 (CD8) were significantly higher in the glutamine cohort than those in the control cohort(SMD = 1.21, 0.25, 1.52, 0.99, and 0.77). Levels of inflammatory markers C-Reactive Protein (CRP), Tumor Necrosis Factor-alpha (TNF-α), Interleukin-1 (IL-1), Interleukin-6 (IL-6), and Interleukin-8 (IL-8) were markedly lower in the glutamine group (SMD = -2.03, -3.29, -1.86, -2.20, and -2.29). The glutamine cohort also exhibited a significant decrease in liver function indicators Alanine Aminotransferase (ALT) and Aspartate Aminotransferase (AST) (SMD = -1.03 and -1.05), while Cholinesterase (CHE) levels were significantly elevated (SMD = 0.61). Serum protein levels, including Albumin (ALB), Prealbumin (PA), and Total Protein (TP), were significantly higher in patients receiving glutamine (SMD = 0.68, 1.09, and 0.81). In addition, Adverse Events (AEs), defined as overall postoperative complications, were significantly less frequent in the glutamine group (RR = 0.59, 95% CI 0.44‒0.79).

**Conclusion:**

Glutamine supplementation in postoperative hepatic surgery patients enhances immune function, reduces inflammation, improves liver function, and decreases the incidence of AEs.

## Highlights

• Glutamine supplementation increases postoperative IgA, IgM, CD3, and CD4 levels.
• Glutamine reduces CRP, TNF-α, IL-1, IL-6, and IL-8 levels after liver surgery.
• Glutamine decreases ALT and AST, and increases CHE, ALB, PA, and TP levels postoperatively.
• Glutamine decreases overall postoperative complications and adverse events.
• Meta-analysis of 18 RCTs shows consistent benefits of perioperative glutamine use.

## Introduction

Hepatic surgery is a vital therapeutic intervention for managing benign and malignant hepatic tumors, complications associated with liver cirrhosis, and other liver-related conditions.^1^^,^^2^ However, as the liver serves as the central organ for nutrient and substance metabolism, postoperative patients often experience varying degrees of hepatic dysfunction, rendering them susceptible to complications such as liver insufficiency, postoperative infections, and malnutrition.^3^^,^^4^ The concept of Enhanced Recovery After Surgery (ERAS) highlights nutritional support as a key measure to facilitate postoperative recovery, reduce mortality, and decrease the incidence of postoperative complications.^5^ With the increasing depth of research on nutritional support in surgical settings, the concept of immunonutrition has emerged, emphasizing the use of specific nutrients to regulate the immune system and promote recovery.^6^

Glutamine, a conditionally essential amino acid in humans, plays critical roles in modulating immune function, maintaining intestinal mucosal integrity, and facilitating antioxidant responses.^7^^,^^8^ Studies have demonstrated that glutamine supplementation may enhance intestinal permeability, attenuate oxidative stress or inflammatory responses, and improve organ function.^9^^,^^10^ Nevertheless, the efficacy of glutamine supplementation in those who received hepatic surgery remains inconclusive. A cohort study by Chen et al.^11^ suggests that glutamine can alleviate acute inflammatory responses, improve liver function and lymphocyte counts, reduce infectious complications, shorten hospital stays, and enhance prognoses in patients undergoing hepatectomy. Conversely, other studies have reported no significant improvements in liver or immune function with glutamine supplementation.^12^, ^13^, ^14^, ^15^

Recent evidence indicates that anabolic agents, such as oxandrolone, can improve metabolic and inflammatory responses in hypercatabolic states.^16^ In parallel, gut microbiome-targeted interventions, including probiotics and synbiotics, have demonstrated beneficial effects in critically ill patients.^17^ Within this broader therapeutic landscape, glutamine has attracted particular interest because of its dual function as a key metabolic substrate and immunonutrient, especially in supporting intestinal barrier integrity and immune competence following hepatectomy. A meta-analysis has further shown that perioperative nutritional supplementation in liver transplantation, including glutamine, is associated with reduced infectious complications and shorter postoperative hospital stays. Nevertheless, evidence regarding the clinical benefits of glutamine supplementation in patients undergoing hepatectomy remains inconclusive. To address this gap, the present study aims to evaluate the efficacy and safety of perioperative glutamine supplementation in patients undergoing liver surgery. Based on Randomized Controlled Trials (RCTs), this study further assesses whether perioperative glutamine supplementation, compared with non-supplementation, improves postoperative clinical outcomes, including immune and inflammatory markers as well as postoperative complications. This study clarifies the efficacy and safety of glutamine supplementation in the management of the foregoing patients, providing robust evidence-based guidance for the development of more scientific and rational perioperative nutritional support strategies.

## Materials and methods

### Registration

This study followed the PRISMA guidelines.^18^ The protocol was pre-registered on the international systematic review registration platform PROSPERO (Registration n° CRD42024543571).

### Literature search

Relevant literature was identified by searching the following databases: CNKI, VIP, Wanfang Data, PubMed, Embase, Cochrane Library, SinoMed, as well as Web of Science. We utilized the keywords “transplantation”, “resection”, “surgery”, “operation”, “preoperative”, “postoperative”, “cancer”, “liver”, and “glutamine”. The search timeframe covered all publications from database inception to July 31, 2025, without restrictions on language or other filters. (Appendix 1).

### Literature screening

After duplicates were automatically ostracized, two researchers independently screened the articles. Potentially eligible studies were identified in accordance with eligibility criteria. Full texts of these studies were then reviewed to determine eligible studies. Based on the PICOS framework, the inclusion criteria were: 1) Population: Patients who underwent liver surgery; 2) Intervention: Treatment regimens incorporating glutamine supplementation; 3) Comparison: Standard treatment protocols without glutamine supplementation; 4) Outcomes: Immune markers, inflammatory markers, liver function indices, protein levels, and Adverse Events (AEs); 5) Study Design: RCTs. Exclusion criteria were: 1) Inappropriate study types: Reviews, systematic evaluations, conference abstracts, or books; 2) Incomplete outcome descriptions; 3) Duplicate publications; 4) Non-Chinese and non-English articles. Two authors (Meng and Zhang) independently screened records and extracted data, including study characteristics and key findings. Dissents were addressed via discussion with a third author (Wang) to achieve consensus.

### Risk of bias assessment

The risk of bias for each included study was assessed via the Risk of Bias 2.0 (RoB 2.0) tool. Each study was evaluated across five domains: randomization process, deviations from intended interventions, missing outcome data, measurement of outcomes, and selective reporting. Overall risk of bias was categorized as “low risk,” “high risk,” or “some concerns.” Risk of bias assessments were conducted independently by Meng and Zhang, with disagreements resolved through consultation with Wang (Appendix 2).

### Data extraction

Data were extracted independently by two researchers and consolidated by a third researcher. Extracted information encompassed basic study details: author, region, year of publication, and study design (sample size, participant demographics, intervention details, sort of surgery). Outcome measures primarily consisted of immune markers, inflammatory markers, liver function indices, protein levels, and AEs. To ensure consistency and reproducibility of data extraction, a standardized data extraction form was designed per the Cochrane Handbook and PRISMA guidelines, and validated on 10% of included studies.

### Statistical analysis

Statistical analyses were carried out via the meta package 7.0-0 in R 4.4.1. Outcome measures reported in at least two independent sources of evidence were pooled. To account for variations in measurement methods or units across studies, continuous outcome variables were analyzed through the Standardized Mean Difference (SMD), whereas categorical data were examined with Risk Ratio (RR) with corresponding 95% Confidence Intervals (95% CIs) as effect measures. Heterogeneity across studies was evaluated through the *I*^2^ statistic. If *I*^2^ ≥ 50% and p < 0.05, heterogeneity was deemed significant, and a random-effects model was utilized; otherwise, a fixed-effects model was leveraged. Regarding outcomes with significant heterogeneity, sensitivity analyses were undertaken to evaluate the stability of eligible studies. This analysis involved excluding individual studies sequentially and recalculating the pooled effect sizes. If substantial changes in effect size were observed after excluding a study, that study was considered a potential source of heterogeneity. Conversely, if excluding any single study did not significantly alter the pooled effect size, the included studies were considered stable. Subgroup analyses were performed by the sort of liver surgery (liver transplantation or hepatectomy). For outcome measures reported in more than two studies, publication bias was assessed using funnel plots and Egger’s regression test. Funnel plot asymmetry or an Egger’s p < 0.05 denotes publication bias. For outcomes exhibiting substantial heterogeneity (*I*^2^ > 50%), subgroup analyses were conducted to explore potential sources of heterogeneity, including glutamine dosage, formulation, duration of administration, and timing of outcome assessment.

## Results

### Literature retrieval

An initial search identified 2466 articles. After titles and abstracts were reviewed, 2400 were ostracized, leaving 66 for full-text screening. Subsequently, 47 were removed for the reasons below: 21 failed to satisfy the criteria for intervention, 16 did not match the study design, and 10 were not aligned with the study population. Notably, although Wang (2006) and Qiu (2009) represented the same study, four articles were published with different outcome measures. Consequently, 19 articles from 18 studies were ultimately eligible for meta-analysis (Appendix 3).

### Quality and basic information of included literature

This study incorporated 18 RCTs^5^^,^^14^^,^^15^^,^^19^, ^20^, ^21^, ^22^, ^23^, ^24^, ^25^, ^26^, ^27^, ^28^, ^29^, ^30^, ^31^, ^32^, ^33^, ^34^ from China, Brazil, and India, encompassing a total of 1198 patients. Among them, 599 were in the experimental cohort, and 599 were in the control cohort, with an average age range of 16 to 90. The surgical procedures included liver transplantation and liver resection, while the intervention involved the postoperative administration of glutamine supplementation. Outcome indicators included: 1) Immune Factors: Immunoglobulin A (IgA), Immunoglobulin G (IgG), Immunoglobulin M (IgM), Cluster of Differentiation 3 (CD3), Cluster of Differentiation 4 (CD4), Cluster of Differentiation 8 (CD8), and the CD4/CD8 ratio; 2) Inflammatory Factors: C-Reactive Protein (CRP), Tumor Necrosis Factor-alpha (TNF-α), Interleukin-1 (IL-1), Interleukin-6 (IL-6), and Interleukin-8 (IL-8); 3) Liver Function Indicators: Alanine Aminotransferase (ALT), Aspartate Aminotransferase (AST), Cholinesterase (CHE), Total Bilirubin (TBIL), and Direct Bilirubin (DBIL); 4) Protein Levels: Albumin (ALB), Prealbumin (PA), and Total Protein (TP); 5) AE (Table 1) and Appendix 4.

**Table 1:** Basic information of included studies.

Author	Year	Region	Sample Size	Gender (Male/ Female)	Average Age	Surgical Procedure	Intervention
Yao et al.^5^	2021	China	40	32/8	54.51±8.52	LR	Nutritional Support + Ala-Gln
			40	34/6	53.89±7.91		Nutritional Support
Tong et al.^19^	2021	China	24	14/10	45.10±9.30	LTx	Metronidazole + Gln
			24	16/8	46.10±7.50		Metronidazole
Wang et al.^20^	2018	China	51	34/17	63.46±6.25	LR	Gln
			50	32/18	62.17±5.08		Normal Saline
Gao et al.^21^	2018	China	52	36/16	36.24±18.12	LR	Enteral Nutrition + Ala-Gln
			52	32/20	34.33±17.28		Enteral Nutrition
Li et al.^22^	2019	China	53	32/21	62.3 ± 8.2	LR	PN + Ala-Gln
			53	34/19	63.8 ± 7.1		PN
Wan et al.^23^	2008	China	25	13/12	46.25±14.32	LTx	TPN+Gln
			25	14/11	42.92±13.82		TPN
Zhu et al.^24^	2020	China	40	10/30	19‒85	LR	Intravenous Nutrition + Ala-Gln
			40	20/20	18‒80		Intravenous Nutrition
Tian et al.^25^	2012	China	21	12/9	49.74±14.23	LR	PN + Ala-Gln
			21	15/6	51.57±13.94		PN
Wu et al.^26^	2011	China	60	37/23	56.6 ± 14.4	LR	PN + Gln
			60	41/19	57.8 ± 16.0		Intravenous Nutrition + Ala-Gln
Qin et al.^27^	2011	China	40	47/33			Intravenous Nutrition
			40	47/33			PN
Wang et al.^28^	2006	China	12	6/6			TPN+Ala-Gln
			12	9/3	47.92±12.817		TPN
Wang et al.^30^	2006	China	12	6/6	51.75±8.465	42‒65	LR
			12	9/3	47.92±12.817	42‒65	TPN
Lin et al.^31^	2017	China	15	7/8	50.89±3.89	51.75±8.465	LTx
			15	8/7	50.97±3.31		Sodium Chloride
Wang et al.^14^	2020	China	100	60/40	16‒88	LR	PN + L-Alanylgutamine
			100	65/35	15‒90		PN
Jiang et al.^32^	2007	China	13	/	44.9 ± 11.1	LTx	Enteral Nutrition + Gln Dipeptide
			13	/	44.3 ± 8.0		Enteral Nutrition
Barros et al.^33^	2015	Brazil	16	/	33.8 ± 5.1	LTx	L-Alanylgutamine
			17	/	40.8 ± 3.8		Normal Saline
Qiu et al.^34^	2009	China	22	12/10	50.75±10.47	LTx	TPN+Gln
			22	14/8	45.92±11.82		TPN
Richard et al.^15^	2014	India	11	6/5	45+11	LTx	EN+Gln
			11	5/6	47+11		EN

The ROB2 scale was used to evaluate the quality of 19 articles. Overall, the quality of evidence was judged to be moderate. One study (Barros et al., 2015) was rated as low risk of bias, whereas the remaining studies were classified as having some concerns. The primary reasons for this rating included insufficient reporting of randomization and blinding procedures, as well as the absence of accessible clinical trial registration protocols, which precluded a reliable assessment of selective outcome reporting.

## Statistical analysis results

### Outcome indicator analysis

#### Immune factors

##### Immunoglobulins

**Fig. 1:**
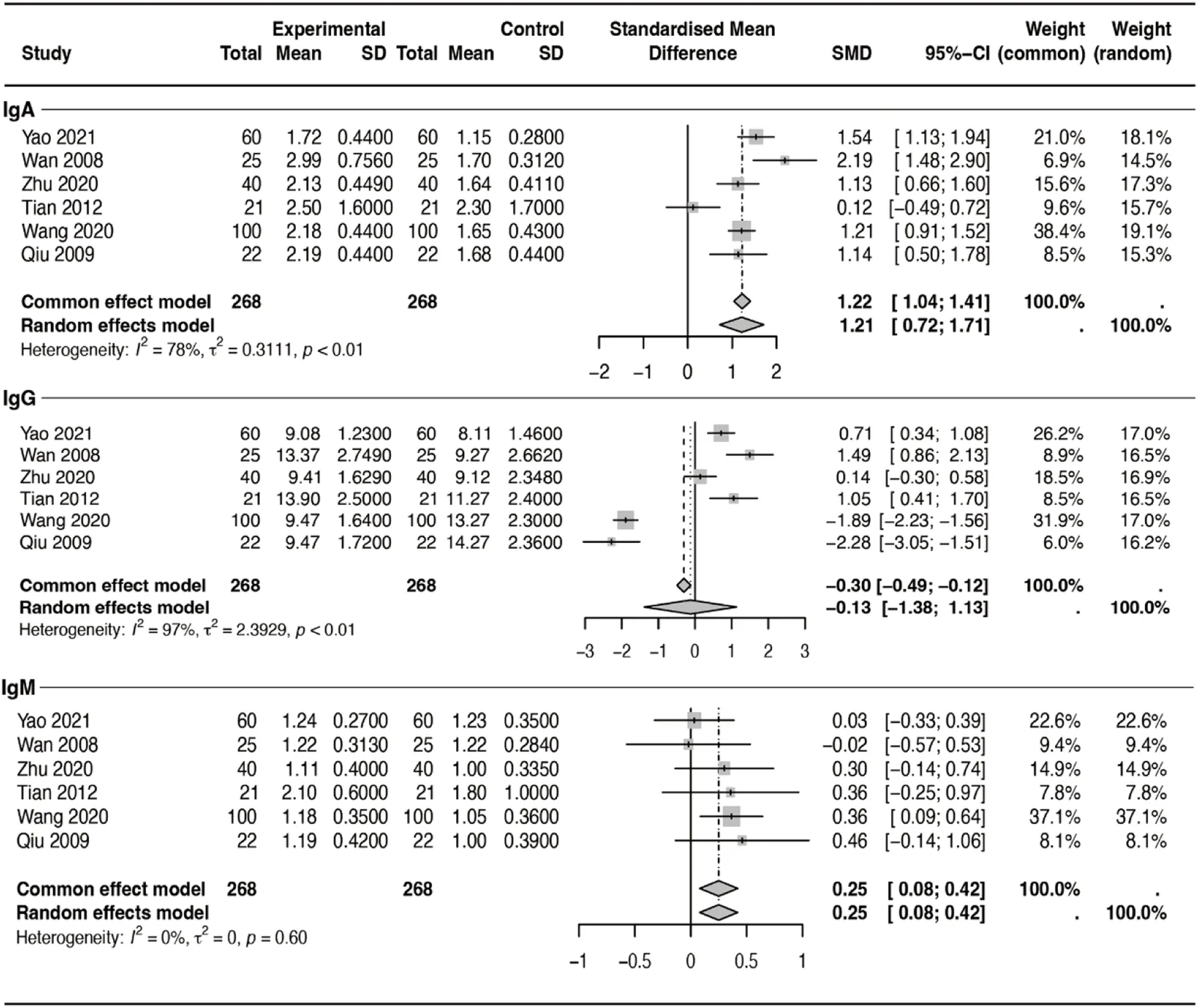
Forest plot of the meta-analysis on the impact of glutamine supplementation on immunoglobulin levels in postoperative liver surgery patients. Note: Immunoglobulin A (IgA), Immunoglobulin M (IgM) and Immunoglobulin G (IgG), Pooled Standardized Mean Difference (SMD) with its 95% Confidence Interval (95% CI).

Six studies^11^^,^^19^, ^20^, ^21^^,^^30^^,^^35^ were included, involving 536 patients, reporting on IgA, IgG, and IgM levels. The meta-analysis revealed that IgA and IgM levels were significantly higher in the experimental cohort than in the control cohort (SMD = 1.21, 95% CI 0.72 to 1.71, p < 0.01; SMD = 0.25, 95% CI 0.08 to 0.42, p < 0.01). There was no notable variance in IgG levels with no statistically significant difference (SMD = −0.13, 95% CI −1.38 to 1.13, p = 0.85) (Fig. 1).

##### T-cell subsets

**Fig. 2:**
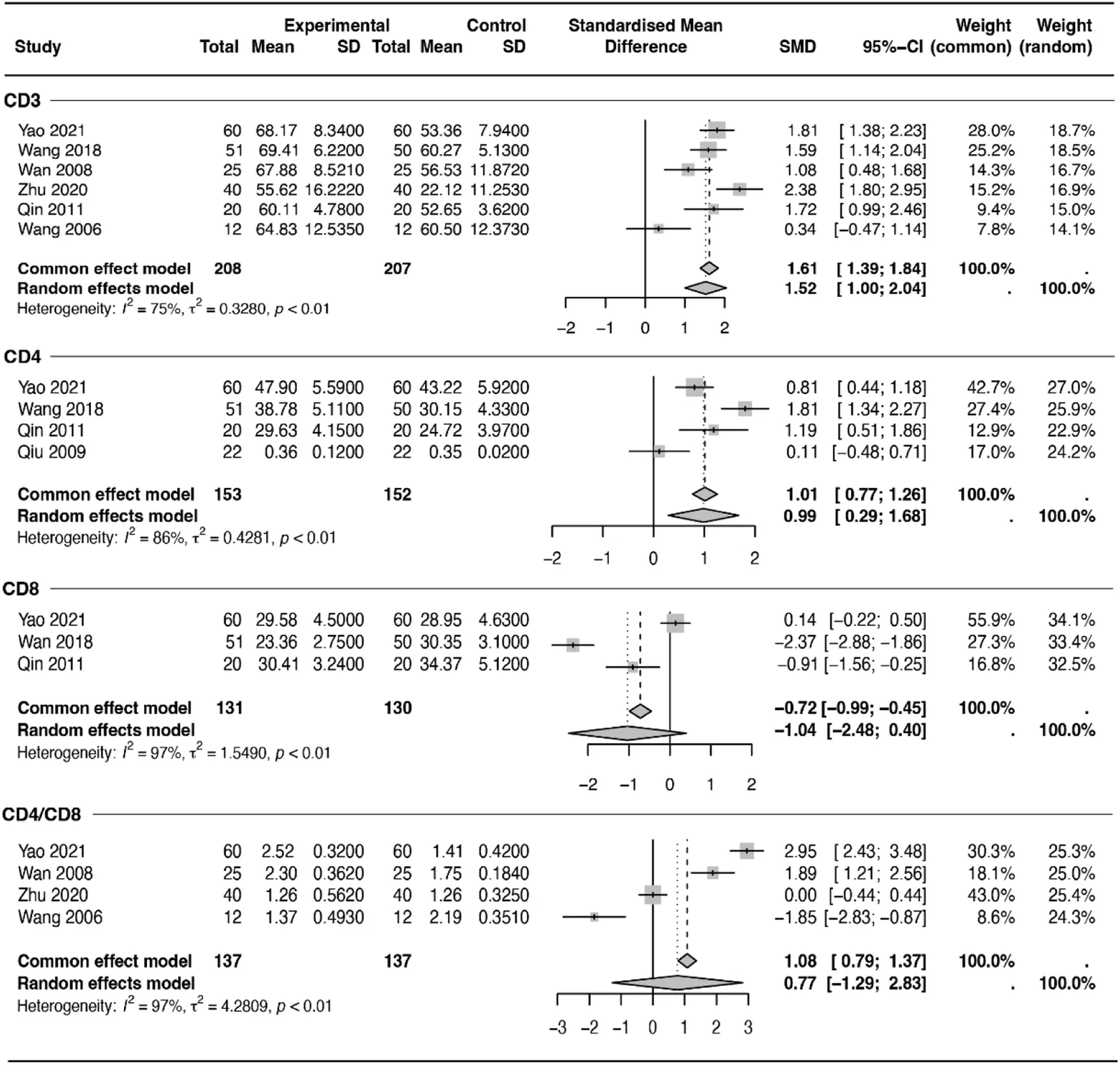
Forest plot of the meta-analysis on the impact of glutamine supplementation on T-cell subtypes in postoperative liver surgery patients. Note: Cluster of Differentiation 3 (CD3), Cluster of Differentiation 4 (CD4), Cluster of Differentiation 8 (CD8), and the CD4/CD8 ratio, Pooled Standardized Mean Difference (SMD) with its 95% Confidence Interval (95% CI).

Six studies^14^^,^^19^^,^^20^^,^^23^^,^^26^^,^^35^ reported CD3 levels in 415 patients. Four studies^14^^,^^23^^,^^30^^,^^35^ reported CD4 levels in 305 patients. Three studies^19^^,^^23^^,^^35^ reported CD8 levels in 261 patients, and four studies^19^^,^^20^^,^^26^^,^^35^ reported CD4/CD8 ratios in 274 patients. According to the meta-analysis, the experimental group’s CD3/CD4 ratios were noticeably greater than those of the control cohort (SMD = 1.52, 95% CI 1.00 to 2.04, p < 0.01; SMD = 0.99, 95% CI 0.29 to 1.68, p < 0.01). The result for CD8 levels compared with the control group showed (SMD = −1.04, 95% CI −2.48‒0.4, p = 0.16), and the result for CD4/CD8 levels compared with the control group showed (SMD = 0.77, 95% CI −1.29‒2.82, p = 0.46) (Fig. 2). Owing to the extremely wide CIs, the available evidence is insufficient to determine whether glutamine exerts beneficial or harmful effects on CD8⁺ cell counts or the CD4⁺/CD8⁺ ratio.

#### Inflammatory factors

**Fig. 3:**
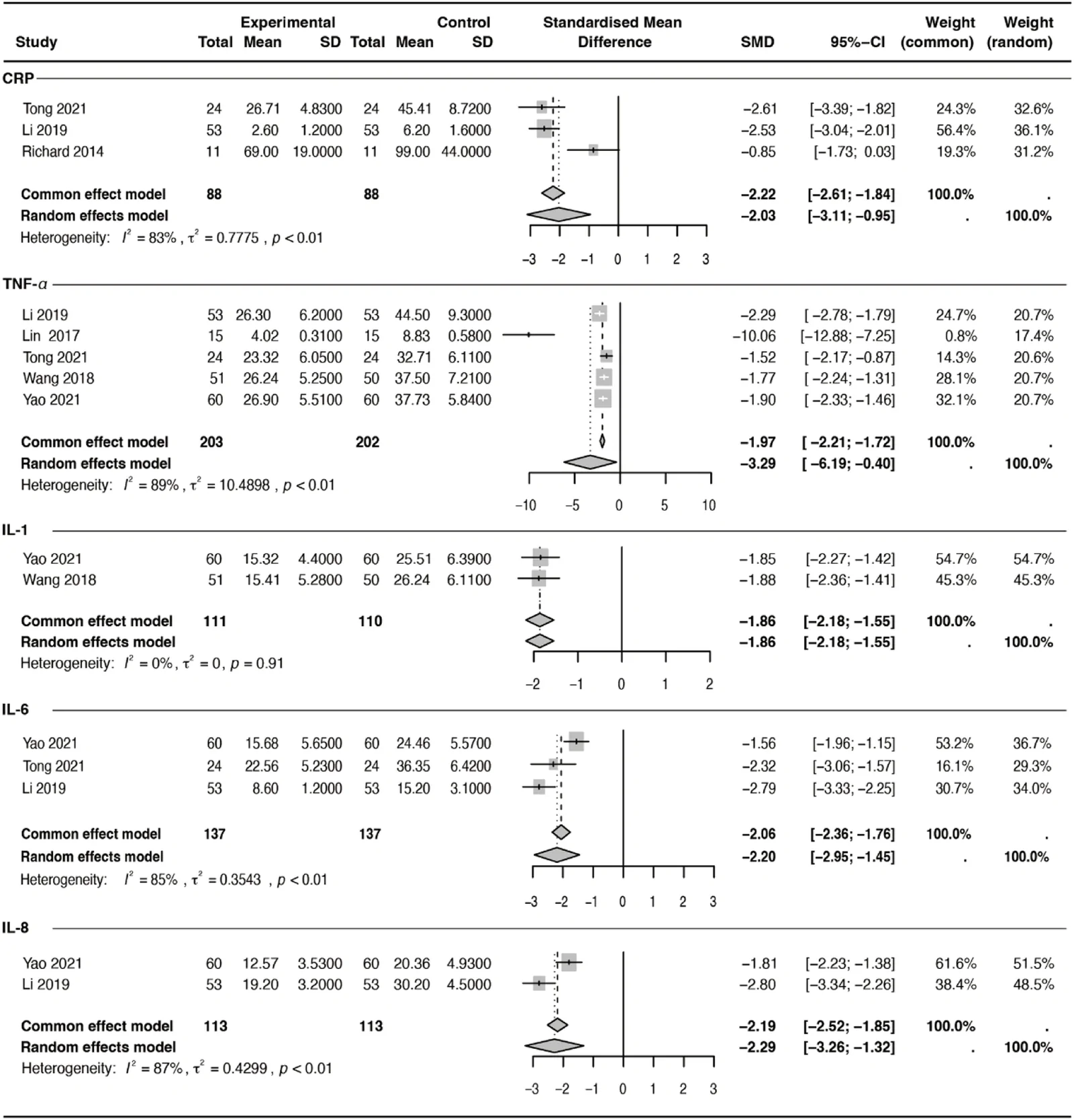
Forest plot of the meta-analysis on the impact of glutamine supplementation on inflammatory cytokines in postoperative liver surgery patients. Note: C-Reactive Protein (CPR), Tumor Necrosis Factor alpha (TNF-α), Interleukin-1 (IL-1), Interleukin-6 (IL-6), and Interleukin-8 (IL-8), Pooled Standardized Mean Difference (SMD) with its 95% Confidence Interval (95% CI).

Three studies^12^^,^^13^^,^^18^ reported CRP levels in 176 patients. Five studies^13^^,^^14^^,^^18^^,^^27^^,^^35^ reported TNF-α levels in 405 patients. Two studies^14^^,^^35^ reported IL-1 levels in 221 patients. Three studies^13^^,^^18^^,^^35^ reported IL-6 levels in 274 patients, and two studies^18^^,^^35^ reported IL-8 levels in 226 patients. The meta-analysis indicated that CRP, TNF-α, IL-1, IL-6, and IL-8 levels were markedly lower in the experimental cohort than in the control cohort (SMD = −2.03, 95% CI −3.11 to −0.95, p < 0.01; SMD = −3.29, 95% CI −6.19 to −0.40, p = 0.03; SMD = −1.86, 95% CI −2.18 to −1.55, p < 0.01; SMD = −2.20, 95% CI −2.95 to −1.45, p < 0.01; SMD = −2.29, 95% CI −3.26 to −1.32, p < 0.01) (Fig. 3).

#### Liver function indicators

**Fig. 4:**
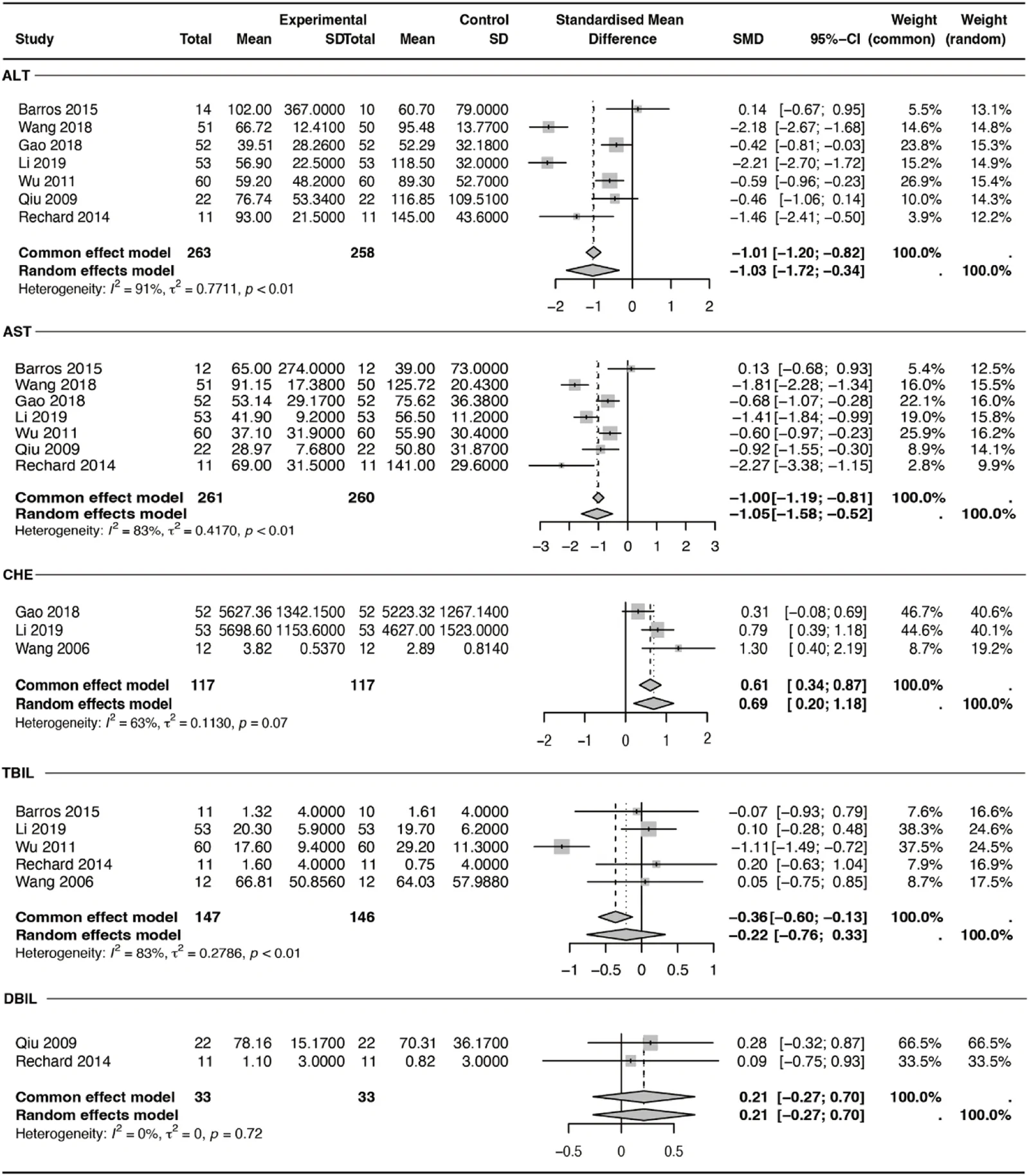
Forest plot of the meta-analysis on the impact of glutamine supplementation on liver function in postoperative liver surgery patients. Note: Aspartate Aminotransferase (AST), Alanine Aminotransferase (ALT), Cholinesterase (CHE), Total Bilirubin (TBIL) and Direct Bilirubin (DBIL), Pooled Standardized Mean Difference (SMD) with its 95% Confidence Interval (95% CI).

Seven studies,^12^^,^^14^^,^^15^^,^^18^^,^^22^^,^^29^^,^^30^ encompassing 521 patients, reported on ALT and AST levels. Three studies^15^^,^^18^^,^^24^ involving 234 patients provided data on CHE levels. Five studies^12^^,^^18^^,^^22^^,^^24^^,^^29^ on 293 patients reported on TBIL levels, and two studies^12^^,^^20^ involving 66 patients focused on DBIL levels. In contrast, patients in the glutamine group exhibited significantly lower ALT and AST levels compared with controls (SMD = −1.03, 95% CI −1.72 to −0.34, p < 0.01; SMD = −1.05, 95% CI −1.58 to −0.52, p < 0.01). Serum CHE levels were significantly higher in the glutamine group than in the control group (SMD = 0.69, 95% CI: 0.20 to 1.18, p < 0.01). No statistically significant differences were observed between groups in TBIL or DBIL levels (SMD = −0.22, 95% CI −0.76 to 0.33, p = 0.44; SMD = 0.21, 95% CI −0.27 to 0.70, p = 0.70; Fig. 4).

#### Protein levels

**Fig. 5:**
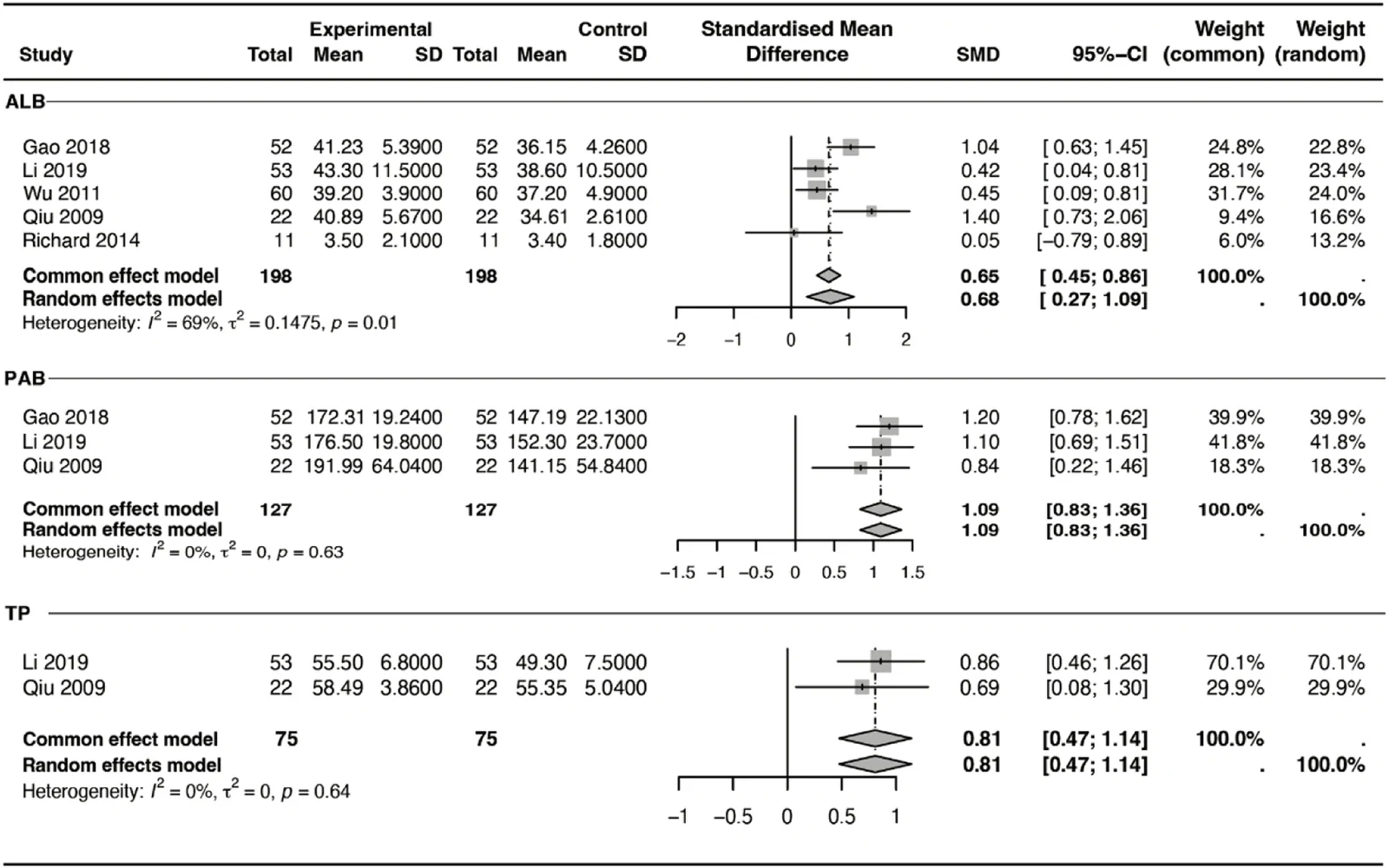
Forest plot of the meta-analysis on the impact of glutamine supplementation on protein levels in postoperative liver surgery patients. Note: Albumin (ALB), Prealbumin (PAB), Cholinesterase (CHE) and Total Protein (TP), Pooled Standardized Mean Difference (SMD) with its 95% Confidence Interval (95% CI).

Five studies^12^^,^^15^^,^^18^^,^^22^^,^^30^ involving 396 patients reported on ALB levels. Three studies^15^^,^^18^^,^^30^ on 254 patients provided data on PA levels, while two studies,^18^^,^^30^ involving 150 patients, focused on TP levels. It was found that the experimental cohort had lower levels of ALB, PA, and TP than the control group, with statistically significant differences (SMD = 0.68, 95% CI 0.27 to 1.09, p < 0.01; SMD = 1.09, 95% CI 0.83 to 1.36, p < 0.01; SMD = 0.81, 95% CI 0.47 to 1.14, p < 0.01) (Fig. 5).

#### AE ‒ overall postoperative complications

**Fig. 6:**
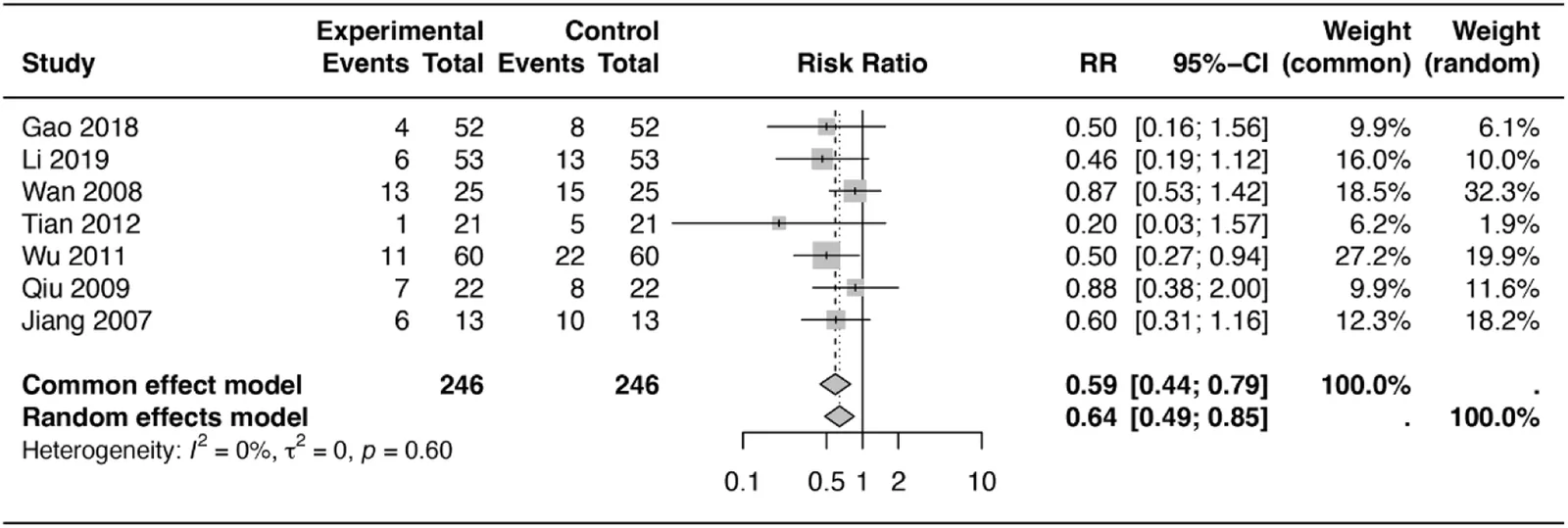
Forest plot of the meta-analysis on the impact of glutamine supplementation on AEs in postoperative liver surgery patients. Note: Overall Postoperative Complications (AE). Pooled Relative Risk (RR) with its 95% Confidence Interval (95% CI).

Seven studies,^12^^,^^15^^,^^18^^,^^19^^,^^21^^,^^22^^,^^28^^,^^30^ involving 492 patients, mentioned the incidence of AEs. Our meta-analysis indicated that the experimental cohort experienced fewer AEs than the control cohort (RR = 0.59, 95% CI 0.44 to 0.79, p < 0.01), suggesting that glutamine may significantly reduce the occurrence of AEs in patients following liver surgery (Fig. 6).

### Postoperative mortality

Four studies^15^^,^^32^, ^33^, ^34^ reported postoperative mortality, involving 125 patients. Two patients in the glutamine group died, and six patients in the control group died. Meta-analysis results showed no statistically significant difference (RR = 0.36, 95% CI 0.08 to 1.62, p > 0.05), as shown in Appendix 5. The current evidence is insufficient to determine whether glutamine supplementation confers beneficial or harmful effects on postoperative mortality.

### Subgroup analysis

Subgroup analysis based on surgery sort revealed that ALT and ALB levels showed significant differences between subgroups, with notable benefits in ALT and ALB changes among patients undergoing liver resection. Changes in IgA, IgG, IgM, CD3, CD4, CD4/CD8, CRP, TNF-α, IL-6, AST, CHE, TBIL, PA, and the incidence of AEs exhibited no significant variations across surgical types (Table 2). Due to the singularity of the surgical types, no subgroup analysis was performed for CD8, IL-1, and IL-8. Additionally, DBIL and TP were not subject to subgroup analysis owing to limited studies (Appendix 6).

**Table 2:** Subgroup analysis results.

Domain	Biomarkers	Liver resection			Liver transplantation			Difference between subgroups
		Trials	Sample Size	Effect Size	Trials	Sample Size	Effect Size	
Inflammatory Markers	CRP	2	128	SMD [95%CI]=−1.73 [−3.37, −0.09]	1	48	SMD [95%CI]=−2.61 [−3.39, −1.82]	p = 0.34
	TNF-α	4	357	SMD [95%CI]=−3.81 [−7.5, −0.11]	1	48	SMD [95%CI]=−1.52 [−2.17, −0.87]	p = 0.23
	IL-6	2	226	SMD [95%CI]=−2.16 [−3.37, −0.95]	1	48	SMD [95%CI]=−2.32 [−3.06, −1.57]	p = 0.83
Immunity	IgA	4	442	SMD [95%CI]=1.03 [0.84, 1.59]	2	94	SMD [95%CI]=1.65 [0.62, 2.68]	p = 0.30
	IgG	4	442	SMD [95%CI]=−0.01 [−1.31, 1.29]	2	94	SMD [95%CI]=−0.39 [−4.09, 3.31]	p = 0.85
	IgM	4	442	SMD [95%CI]=0.26 [0.07, 0.45]	2	94	SMD [95%CI]=0.20 [−0.21, 0.61]	p = 0.83
Liver Function	**ALT**	**5**	**453**	**SMD [95%CI]=−1.36 [−2.13, −0.58]**	**2**	**68**	**SMD [95%CI]=−0.25 [−0.73, 0.24]**	**p=0.02**
	AST	5	453	SMD [95%CI]=−1.26 [−1.85, −0.68]	2	68	SMD [95%CI]=−0.43 [−1.46, 0.60]	p = 0.17
	CHE	2	210	SMD [95%CI]=0.55 [0.07, 1.02]	1	224	SMD [95%CI]=1.30 [0.40, 2.19]	p = 0.15
	TBIL	3	248	SMD [95%CI]=−0.01 [−0.59, 0.58]	2	45	SMD [95%CI]=−0.31 [−1.16, 0.55]	p = 0.57
Protein	**ALB**	**4**	**352**	**SMD [95%CI]=0.55 [0.19, 0.91]**	**1**	**44**	**SMD [95%CI]=1.40 [0.73, 2.06]**	**p=0.03**
	PAB	2	210	SMD [95%CI]=1.15 [0.86, 1.44]	1	44	SMD [95%CI]=0.84 [0.22, 1.46]	p = 0.37
AE	AE	4	372	SMD [95%CI]=0.46 [0.29, 0.72]	3	120	SMD [95%CI]=0.79 [0.55, 1.13]	p = 0.08

### Sensitivity analysis

Due to the limited number of eligible studies for IL-1, IL-8, DBIL, and TP (only two studies each), sensitivity analyses were not performed. For IgM, CHE, PA, and AE, no heterogeneity was observed (*I*^2^ ≤ 50%, p > 0.05); therefore, sensitivity analyses were also not conducted. Sensitivity analyses for the remaining outcomes were performed by sequentially excluding individual studies. Specifically, for the CRP outcome, after excluding the study by Richard (2014),^15^ a significant difference was observed (SMD = −2.55, 95% CI −2.98 to −2.12, p < 0.01, *I*^2^ = 0%). For the TNF-α outcome, exclusion of the study by Lin (2017) resulted in a significant difference in the meta-analysis (SMD = −1.90, 95% CI −2.17 to −1.63, p < 0.01, *I*^2^ = 24%). For CHE, removal of the study by Li (2019) led to a notable change in the pooled result (SMD = 0.72, 95% CI −0.24 to 1.67, p = 0.14, *I*^2^ = 75%). For the CD4 outcome, exclusion of the study by Qin (2011) also produced a marked change in the meta-analysis results (SMD=0.92, 95% CI −0.03 to 1.87, p = 0.06, *I*^2^ = 91%). For CD8, exclusion of the study by Yao (2021) resulted in substantial variation in the pooled estimate (SMD = −1.65, 95% CI −3.09 to −0.22, p = 0.02, *I*^2^ = 92%). These findings indicate that, after excluding the studies by Lin (2017), Li (2019), Qin (2011), and Yao (2021), the results for TNF-α, CHE, CD4, and CD8 showed opposite trends, suggesting that differences in treatment duration and formulations in these studies may have influenced the overall outcomes.

### Heterogeneity test and publication bias test

For outcomes with substantial heterogeneity (*I*^2^ > 50%), subgroup analyses were performed to explore potential sources of heterogeneity. Treatment duration was identified as the primary source of heterogeneity for IgA and CHE. The timing of outcome assessment contributed to heterogeneity in CD3 outcomes. Glutamine dosage was the main source of heterogeneity for CD4, CD8, and ALB, while differences in formulation (oral vs. intravenous) accounted for heterogeneity in CRP. No clear sources of heterogeneity were identified for IgG, CD4/CD8 ratio, IL-6, ALT, AST, or TBIL. Publication bias was assessed using funnel plots and Egger’s regression test for outcomes with fewer than 10 included studies. Egger’s test could not be performed for IL-1, IL-8, DBIL, or TP because only two studies were available. For all remaining outcomes, Egger’s test yielded p-values > 0.05, indicating no evidence of significant publication bias (Appendix 8 and Appendix 9).

## Discussion

This meta-analysis suggests that glutamine supplementation may confer multiple postoperative benefits in patients undergoing liver surgery. Specifically, glutamine was associated with improvements in immune function, as evidenced by increased levels of IgA, IgM, CD3, and CD4; reductions in inflammatory mediators, including CRP, TNF-α, IL-1, IL-6, and IL-8; attenuation of liver injury, reflected by decreased AST and ALT levels alongside increased CHE levels; enhancement of nutritional status through elevated ALB, PA, and TP; and a reduced risk of postoperative complications. Meanwhile, no statistical correlation was found between intraoperative glutamine supplementation and postoperative mortality. Our findings align with emerging evidence supporting metabolic modulation as a key mechanism in surgical recovery, while underscoring the need for dose standardization in glutamine supplementation.^16^

Subgroup analyses informed by sensitivity and heterogeneity assessments further clarified effect modifiers. Drug formulation was identified as the source of heterogeneity for CRP outcomes: the study by Richard et al. (2014) administered oral glutamine, whereas Tong et al. (2021) and Li et al. (2019) used intravenous formulations. Treatment duration emerged as the source of heterogeneity for CHE outcomes, with Gao et al. (2018) administering glutamine for 6‒8 days, compared with a fixed 7-day regimen in Li et al. (2019) and Wang et al. (2006). These findings were consistent with sensitivity analyses, suggesting that both the route of administration and duration of treatment may meaningfully influence therapeutic efficacy. For TNF-α, a large effect size was initially observed. Subgroup analysis identified treatment duration as the principal source of heterogeneity. Unlike studies administering intravenous glutamine for 7-days (e.g., Li et al., 2019), Lin et al. (2017) used a single intraoperative bolus dose of 0.75 g/kg, resulting in a more pronounced reduction in TNF-α. After exclusion of Lin et al. (2017), the pooled effect remained significant with substantially reduced heterogeneity (SMD = −1.90, 95% CI: −2.17 to −1.63; *I*^2^ = 24.3%), reinforcing the conclusion that glutamine supplementation reduces TNF-α levels following liver surgery.

Mechanistically, glutamine is critical in postoperative immune regulation in patients with liver disease by enhancing immune cell activity and increasing immunoglobulin synthesis.^36^ Following hepatectomy or liver transplantation, glutamine can upregulate antioxidant systems, including Heat Shock Proteins (HSPs) and Glutathione (GSH), suppress proinflammatory cytokine release (e.g., TNF-α), and reduce mitochondrial Reactive Oxygen Species (ROS) generation, thereby significantly improving liver function.^8^ Notably, while inhibition of glutamine uptake and metabolism has demonstrated therapeutic potential in oncology settings),^37^^,^^38^ glutamine supplementation remains widely used as an adjunctive clinical intervention in trauma, burns, fractures, infection, sepsis, malignancy, acute pancreatitis, perioperative care, and critically ill patients. Through enhancement of immune competence, glutamine supplementation has been associated with improved clinical outcomes across these contexts.^39^ Our findings suggest that glutamine supplementation significantly elevates levels of IgA, IgM, CD3, and CD4. This confirms that glutamine supplementation can significantly improve immune status in patients after liver surgery.

Glutamine also effectively reduces inflammation. As a main energy source for lymphocytes, macrophages, intestinal epithelial cells, and other cells, glutamine can reduce intestinal mucosal atrophy, maintain intestinal barrier function, and promote the growth of the intestinal microbiota, thereby lowering the risk of intestinal infections. These effects contribute to the suppression of systemic inflammatory responses.^40^ Additionally, glutamine regulates the production and release of inflammatory mediators, decreasing TNF-α, IL-6, and IL-8 expression, alleviating oxidative stress, and lowering surgical infection risk.^41^^,^^42^ For liver resection patients, glutamine promotes liver regeneration by enhancing energy supply to hepatocyte proliferation and inhibiting bacterial and endotoxin translocation, thus strengthening immune function and reducing inflammatory responses.^5^ Our study indicates that glutamine supplementation significantly reduces CRP, TNF-α, IL-1, IL-6, and IL-8 levels, with notable improvements in CRP, TNF-α, and IL-6 levels in those having received liver resection. These results confirm that glutamine can substantially diminish inflammatory responses in patients following liver surgery.

Furthermore, glutamine effectively alleviates liver injury in post-operative patients. It increases the synthesis of reduced GSH in hepatocyte mitochondria, thereby blocking the vicious cycle caused by free radicals, enhancing the antioxidant capacity of the body, and protecting liver function.^20^ ALT and AST are widely used in liver function assessment after liver surgery, and their levels are influenced by various factors such as surgery duration, extent of liver resection, and blood flow occlusion.^43^, ^44^, ^45^ ALT and AST are primarily localized in the cytosol and mitochondria of hepatocytes, and their postoperative elevation reflects hepatocellular injury caused by surgical trauma, ischemia-reperfusion injury, or inflammatory responses. Glutamine protects hepatocytes by enhancing GSH synthesis, suppressing mitochondrial ROS production, and reducing the release of proinflammatory cytokines,^8^ thereby contributing to the observed reductions in ALT and AST levels. In contrast, elevations in TBIL and DBIL are mainly associated with impaired bilirubin metabolism and biliary excretion.^46^^,^^47^ As glutamine does not directly target the biliary system or bilirubin metabolic pathways, no significant effects on TBIL or DBIL were observed. Our findings reveal that glutamine supplementation significantly lowers ALT, AST, and CHE levels, with a more pronounced effect observed in liver resection patients. This substantiates that glutamine supplementation can notably improve liver function in patients after liver surgery. Glutamine has been shown to effectively elevate protein levels in postoperative liver surgery patients. As one of the most active organs in human protein synthesis, the liver plays a pivotal role in providing essential energy substrates for the body. Following liver surgery, impaired liver function leads to inadequate energy supply. The supplementation of glutamine postoperatively helps provide energy through its metabolic products. Moreover, exogenous glutamine serves as a precursor for pyrimidines, purines, and nucleotides, thereby facilitating the synthesis of proteins and RNA.^22^ Liver dysfunction results in various metabolic disturbances, including a reduced capacity for protein synthesis. TP levels reflect the recovery of liver function after surgery, while PA and ALB levels serve as indicators of postoperative protein synthesis capacity. Low postoperative TP, PA, and ALB levels have been confirmed as independent risk factors for the occurrence of postoperative complications. Our findings demonstrate that glutamine supplementation significantly increases levels of ALB, PA, and TP, with the greatest benefit observed in ALB improvement in liver transplant patients and PA improvement in liver resection patients. These findings suggest that glutamine may significantly increase protein levels in patients undergoing liver surgery. However, the actual clinical effects require further confirmation through well-designed clinical studies.

Glutamine also effectively reduces the occurrence of AEs in postoperative liver patients. Nutritional support is vital in the perioperative treatment of liver surgery patients. Under conditions of illness, poor nutritional status, and other stressors, the body’s demand for glutamine increases, and endogenous synthesis becomes insufficient. Glutamine, an essential amino acid, has multiple beneficial effects, including enhancing immune function, reducing inflammation, improving metabolism, and promoting protein synthesis, which in turn reduces the incidence of bleeding, infection, and other AEs, and accelerates postoperative recovery.^48^ Owing to limited data, it was impossible to perform subgroup analyses according to specific complication types. Nevertheless, the findings consistently indicate that glutamine supplementation reduces the risk of overall postoperative complications.

Previous studies have indicated that glutamine supplementation in people receiving colorectal cancer surgery effectively reduces the likelihood of postoperative complications, promotes the recovery of intestinal function, and increases serum ALB levels.^49^ The application of glutamine in the critically ill population has also been proven to ameliorate overall body function and reduce the incidence of complications and adverse reactions.^50^^,^^51^ These findings suggest that glutamine holds potential as a supportive measure in major surgeries, including liver resection or transplantation. This study is the first to systematically collect existing literature and conduct a meta-analysis to comprehensively examine the effects of glutamine on postoperative liver surgery patients. Furthermore, subgroup analysis suggests potential differences in the effects of glutamine supplementation between liver transplant and liver resection populations.

However, this study also has several limitations. First, subgroup analysis based on different surgical approaches revealed no significant differences in some outcome indicators, which may arise from heterogeneity within all included populations. Second, some outcome indicators exhibited high heterogeneity. For IgG, CD4/CD8 ratio, IL-6, ALT, AST, and TBIL, no definitive sources of heterogeneity were identified. This may be related to the lack of information on preoperative nutritional status in our study population. Therefore, caution is necessary when these findings are applied. Third, most eligible studies were conducted in Asian populations, which may limit the generalizability of the results to other regions due to potential differences in clinical practice patterns, perioperative management, and patient characteristics. Consequently, the conclusions should be cautiously extrapolated to non-Asian populations, and validation through international, multicenter studies is warranted. Fourth, the majority of the studies considered in this research focused solely on the short-term effects of glutamine in post-liver surgery populations; the absence of long follow-up may lead to an incomplete assessment of the issue. Fifth, as a meta-analysis synthesizing previously published studies, this investigation is inherently observational in nature and cannot establish causal relationships. Although several outcomes demonstrated statistically significant differences, the complexity of postoperative immune and metabolic regulation necessitates a cautious approach to the clinical application of glutamine. Moreover, the limited number of included studies for certain outcomes precluded a formal assessment of publication bias, which may increase the risk of overestimating treatment effects. Meanwhile, small-study effects may have further inflated the estimated effect sizes for less frequently reported outcomes.

Future research should prioritize well-designed, adequately powered RCTs with rigorous randomization and blinding procedures, standardized timing of outcome assessment, and comprehensive evaluation of baseline nutritional status. Clear protocols regarding the timing, dosage, route of administration, and duration of glutamine supplementation are also needed. In addition, prospective cohort studies focusing on long-term outcomes may further clarify the sustained effects of glutamine after liver surgery. Subgroup analyses stratified by surgical type revealed no significant differences across most outcomes, suggesting that the effects of glutamine may be relatively consistent across different liver surgery procedures. This finding supports the potential broad applicability of glutamine as a perioperative adjunct. However, the absence of significant subgroup effects may also reflect limited sample sizes and residual heterogeneity. Larger, surgery-specific studies are therefore required to delineate the differential effects of glutamine across surgical contexts.

## Conclusion

This study indicates that glutamine can improve immune function in patients after liver surgery, reduce the levels of specific inflammatory factors to some extent, exert a certain alleviating effect on liver injury, and lower the overall risk of postoperative complications. Nevertheless, given the aforementioned limitations, further large-scale, high-quality RCTs or well-designed cohort studies are necessary to confirm these findings and to guide evidence-based clinical practice.

## Patient and public involvement

Patients and/or the public were not involved in the design, conduct, reporting, or dissemination plans of this research.

## Patient consent for publication

Not applicable.

## Ethics approval

Not applicable.

## Funding

This research received no specific grant from any funding agency in the public, commercial, or not-for-profit sectors.

## Data availability statement

Data are available upon reasonable request.

## CRediT authorship contribution statement

**Xuezheng Meng:** Conceptualization, Methodology, Formal analysis, Investigation, Writing – original draft, Writing – review & editing. **Mingming Zhang:** Resources. **Xinran Wang:** Supervision.

## Conflicts of interest

The authors declare no conflicts of interest.

## Appendix Supplementary materials

Image, application 1

Image, application 2

Image, application 3

Image, application 4

Image, application 5

Image, application 6

Image, application 7

Image, application 8

Image, application 9

Image, application 10

Image, application 11
