# Supplementary material for: Efficacy of glutamine supplementation in postoperative patients undergoing hepatic surgery: a systematic review and meta-analysis of randomized controlled trials
Source: Clinics (Sao Paulo). 2026 Sep 1;81:101086. doi: 10.1016/j.clinsp.2026.101086 (PMC13570316; doi:10.1016/j.clinsp.2026.101086)
Supplement: Supplementary file 1 [file mmc1.docx]

**Appendix 1 S**earch history

| Database | Search History | Search Results | Search Time |
| --- | --- | --- | --- |
| PubMed | | | |
| 1 | liver[Title/Abstract] OR hepatic[Title/Abstract] | 1,117,617 | 2025/7/24 |
| 2 | "Ghost Surgery" OR "operation" OR "operation care" OR "operative intervention" OR "Operative Procedure" OR "operative repair" OR "operative restoration" OR "operative surgery" OR "operative surgical procedure" OR "operative surgical procedures" OR "operative treatment" OR "research surgery" OR "resection" OR "surgery" OR "surgical care" OR "surgical correction" OR "surgical diagnosis" OR "surgical diagnostic techniques" OR "surgical exposure" OR "surgical intervention" OR "surgical management" OR "surgical operation" OR "surgical practice" OR "Surgical Procedure" OR "surgical repair" OR "surgical research" OR "surgical restoration" OR "surgical service" OR "surgical speciality" OR "surgical specialties" OR "surgical specialty" OR "surgical therapy" OR "surgical treatment" OR "preoperative" OR "postoperative" OR "perioperative" | 2,611,795 | 2025/7/24 |
| 3 | liver[MeSH Terms] | 479678 | 2025/7/24 |
| 5 | surgery[MeSH Terms] OR operation[MeSH Terms] | 3649031 | 2025/7/24 |
| 6 | hepatectomy[MeSH Terms] | 35203 | 2025/7/24 |
| 7 | "hemihepatectomy"[Title/Abstract] OR "hepatic resection"[Title/Abstract] OR "hepatic surgery"[Title/Abstract] OR "liver operation"[Title/Abstract] OR "liver resection"[Title/Abstract] OR "liver surgery"[Title/Abstract] | 23544 | 2025/7/24 |
| 8 | Liver Transplantation[MeSH Terms] | 65881 | 2025/7/24 |
| 9 | "Auxiliary Liver Transplantation"[Title/Abstract] OR "Hepatic Graft"[Title/Abstract] OR "Hepatic Transplant"[Title/Abstract] OR "Hepatic Transplantation"[Title/Abstract] OR "Liver Graft"[Title/Abstract] OR "Liver Grafting"[Title/Abstract] OR "Liver heterotopic Transplantation"[Title/Abstract] OR "Liver Orthotopic Graft"[Title/Abstract] OR "Liver Orthotopic Transplantation"[Title/Abstract] OR "Liver Tissue Transplantation"[Title/Abstract] OR "Liver Transplant"[Title/Abstract] OR "Liver Transplantation"[Title/Abstract] OR "Orthotopic Liver Transplantation"[Title/Abstract] | 74203 | 2025/7/24 |
| 10 | glutamine[MeSH Terms] | 20143 | 2025/7/24 |
| 11 | "acutil"[Title/Abstract] OR "adamin G"[Title/Abstract] OR "dl glutamine"[Title/Abstract] OR "D Glutamine"[Title/Abstract] OR "endari"[Title/Abstract] OR "glumin"[Title/Abstract] OR "glutamic acid 5 amide"[Title/Abstract] OR "glutamic acid monoamide"[Title/Abstract] OR "glutamin"[Title/Abstract] OR "glutamine"[Title/Abstract] OR "glutamine residue"[Title/Abstract] OR "kabi glutamine"[Title/Abstract] OR "l glutamin"[Title/Abstract] OR "l glutamine"[Title/Abstract] OR "levo glutamin"[Title/Abstract] OR "levo glutamine"[Title/Abstract] OR "levoglutamide"[Title/Abstract] OR "levoglutamine"[Title/Abstract] OR "L Glutamine"[Title/Abstract] OR "nutrestore"[Title/Abstract] OR "xyndari"[Title/Abstract] | 44127 | 2025/7/24 |
| 12 | (((#1 OR #3) AND (#2 OR #5)) OR (#6 OR #7) OR (#8 OR #9)) AND (#10 OR #11) | 644 | 2025/7/24 |
| Embase | | | |
| 1 | 'liver transplantation'/exp | 146739 | 2025/7/24 |
| 2 | 'auxiliary liver transplantation':ti,ab,kw OR 'hepatic graft':ti,ab,kw OR 'hepatic transplant':ti,ab,kw OR 'hepatic transplantation':ti,ab,kw OR 'liver graft':ti,ab,kw OR 'liver grafting':ti,ab,kw OR 'liver heterotopic transplantation':ti,ab,kw OR 'liver orthotopic graft':ti,ab,kw OR 'liver orthotopic transplantation':ti,ab,kw OR 'liver tissue transplantation':ti,ab,kw OR 'liver transplant':ti,ab,kw OR 'liver transplantation':ti,ab,kw OR 'orthotopic liver transplantation':ti,ab,kw | 126648 | 2025/7/24 |
| 3 | 'hepatectomy'/exp | 74069 | 2025/7/24 |
| 4 | 'hemihepatectomy':ti,ab,kw OR 'hepatic resection':ti,ab,kw OR 'hepatic surgery':ti,ab,kw OR 'liver operation':ti,ab,kw OR 'liver resection':ti,ab,kw OR 'liver surgery':ti,ab,kw | 36250 | 2025/7/24 |
| 5 | 'surgery'/exp OR 'operation'/exp | 6394650 | 2025/7/24 |
| 6 | 'ghost surgery':ti,ab,kw OR 'operation':ti,ab,kw OR 'operation care':ti,ab,kw OR 'operative intervention':ti,ab,kw OR 'operative procedure':ti,ab,kw OR 'operative repair':ti,ab,kw OR 'operative restoration':ti,ab,kw OR 'operative surgery':ti,ab,kw OR 'operative surgical procedure':ti,ab,kw OR 'operative surgical procedures':ti,ab,kw OR 'operative treatment':ti,ab,kw OR 'research surgery':ti,ab,kw OR 'resection':ti,ab,kw OR 'surgery':ti,ab,kw OR 'surgical care':ti,ab,kw OR 'surgical correction':ti,ab,kw OR 'surgical diagnosis':ti,ab,kw OR 'surgical diagnostic techniques':ti,ab,kw OR 'surgical exposure':ti,ab,kw OR 'surgical intervention':ti,ab,kw OR 'surgical management':ti,ab,kw OR 'surgical operation':ti,ab,kw OR 'surgical practice':ti,ab,kw OR 'surgical procedure':ti,ab,kw OR 'surgical repair':ti,ab,kw OR 'surgical research':ti,ab,kw OR 'surgical restoration':ti,ab,kw OR 'surgical service':ti,ab,kw OR 'surgical speciality':ti,ab,kw OR 'surgical specialties':ti,ab,kw OR 'surgical specialty':ti,ab,kw OR 'surgical therapy':ti,ab,kw OR 'surgical treatment':ti,ab,kw OR 'preoperative':ti,ab,kw OR 'postoperative':ti,ab,kw OR 'perioperative':ti,ab,kw | 3549985 | 2025/7/24 |
| 7 | 'glutamine'/exp | 54285 | 2025/7/24 |
| 8 | 'acutil':ti,ab,kw OR 'adamin g':ti,ab,kw OR 'dl glutamine':ti,ab,kw OR 'd glutamine':ti,ab,kw OR 'endari':ti,ab,kw OR 'glumin':ti,ab,kw OR 'glutamic acid 5 amide':ti,ab,kw OR 'glutamic acid monoamide':ti,ab,kw OR 'glutamin':ti,ab,kw OR 'glutamine':ti,ab,kw OR 'glutamine residue':ti,ab,kw OR 'kabi glutamine':ti,ab,kw OR 'l glutamin':ti,ab,kw OR 'levo glutamin':ti,ab,kw OR 'levo glutamine':ti,ab,kw OR 'levoglutamide':ti,ab,kw OR 'levoglutamine':ti,ab,kw OR 'l glutamine':ti,ab,kw OR 'nutrestore':ti,ab,kw OR 'xyndari':ti,ab,kw | 53395 | 2025/7/24 |
| 9 | 'liver'/exp | 742998 | 2025/7/24 |
| 10 | liver:ti,ab,kw OR hepatic:ti,ab,kw | 1539618 | 2025/7/24 |
| 11 | ((#9 OR #10) AND (#5 OR #6) OR #3 OR #4 OR #1 OR #2) AND (#7 OR #8) | 1093 | 2025/7/24 |
| Cochrane |  |  |  |
| 1 | MeSH descriptor: [Liver] explode all trees | 4633 |  |
| 2 | (liver OR hepatic):ti,ab,kw | 78481 |  |
| 3 | MeSH descriptor: [General Surgery] explode all trees | 513 |  |
| 4 | ('Ghost Surgery' OR 'operation' OR 'operation care' OR 'operative intervention' OR 'Operative Procedure' OR 'operative repair' OR 'operative restoration' OR 'operative surgery' OR 'operative surgical procedure' OR 'operative surgical procedures' OR 'operative treatment' OR 'research surgery' OR 'resection' OR 'surgery' OR 'surgical care' OR 'surgical correction' OR 'surgical diagnosis' OR 'surgical diagnostic techniques' OR 'surgical exposure' OR 'surgical intervention' OR 'surgical management' OR 'surgical operation' OR 'surgical practice' OR 'Surgical Procedure' OR 'surgical repair' OR 'surgical research' OR 'surgical restoration' OR 'surgical service' OR 'surgical speciality' OR 'surgical specialties' OR 'surgical specialty' OR 'surgical therapy' OR 'surgical treatment' OR 'preoperative' OR 'postoperative' OR 'perioperative'):ti,ab,kw | 402095 |  |
| 5 | MeSH descriptor: [Hepatectomy] explode all trees | 971 |  |
| 6 | ('hemihepatectomy' OR 'hepatic resection' OR 'hepatic surgery' OR 'liver operation' OR 'liver resection' OR 'liver surgery'):ti,ab,kw | 14779 |  |
| 7 | MeSH descriptor: [Liver Transplantation] explode all trees | 1708 |  |
| 8 | ('Auxiliary Liver Transplantation' OR 'Hepatic Graft' OR 'Hepatic Transplant' OR 'Hepatic Transplantation' OR 'Liver Graft' OR 'Liver Grafting' OR 'Liver heterotopic Transplantation' OR 'Liver Orthotopic Graft' OR 'Liver Orthotopic Transplantation' OR 'Liver Tissue Transplantation' OR 'Liver Transplant' OR 'Liver Transplantation' OR 'Orthotopic Liver Transplantation'):ti,ab,kw | 8232 |  |
| 9 | MeSH descriptor: [Glutamine] explode all trees | 828 |  |
| 10 | ('acutil' OR 'adamin G' OR 'dl glutamine' OR 'D Glutamine' OR 'endari' OR 'glumin' OR 'glutamic acid 5 amide' OR 'glutamic acid monoamide' OR 'glutamin' OR 'glutamine' OR 'glutamine residue' OR 'kabi glutamine' OR 'l glutamin' OR 'l glutamine' OR 'levo glutamin' OR 'levo glutamine' OR 'levoglutamide' OR 'levoglutamine' OR 'L Glutamine' OR 'nutrestore' OR 'xyndari'):ti,ab,kw | 2419 |  |
| 11 | ((#1 OR #2) AND (#3 OR #4) OR #5 OR #6 OR #7 OR #8) AND (#9 OR #10) | 52 |  |
| WOS |  |  |  |
| 28 | TS=((liver OR hepatic) ) | 1247718 |  |
| 32 | TS=(("Ghost Surgery") OR ("operation") OR ("operation care") OR ("operative intervention") OR ("Operative Procedure") OR ("operative repair") OR ("operative restoration") OR ("operative surgery") OR ("operative surgical procedure") OR ("operative surgical procedures") OR ("operative treatment") OR ("research surgery") OR ("resection") OR ("surgery") OR ("surgical care") OR ("surgical correction") OR ("surgical diagnosis") OR ("surgical diagnostic techniques") OR ("surgical exposure") OR ("surgical intervention") OR ("surgical management") OR ("surgical operation") OR ("surgical practice") OR ("Surgical Procedure") OR ("surgical repair") OR ("surgical research") OR ("surgical restoration") OR ("surgical service") OR ("surgical speciality") OR ("surgical specialties") OR ("surgical specialty") OR ("surgical therapy") OR ("surgical treatment") OR ("preoperative") OR ("postoperative") OR ("perioperative”)) | 3534317 |  |
| 39 | TS=(("Auxiliary Liver Transplantation") OR ("Hepatic Graft") OR ("Hepatic Transplant") OR ("Hepatic Transplantation") OR ("Liver Graft") OR ("Liver Grafting") OR ("Liver heterotopic Transplantation") OR ("Liver Orthotopic Graft") OR ("Liver Orthotopic Transplantation") OR ("Liver Tissue Transplantation") OR ("Liver Transplant") OR ("Liver Transplantation") OR ("Orthotopic Liver Transplantation") ) | 122535 |  |
| 41 | TS=(("hemihepatectomy") OR ("hepatic resection") OR ("hepatic surgery") OR ("liver operation") OR ("liver resection") OR ("liver surgery")) | 30384 |  |
| 47 | TS=(("acutil") OR ("adamin G") OR ("dl glutamine") OR ("D Glutamine") OR ("endari") OR ("glumin") OR ("glutamic acid 5 amide") OR ("glutamic acid monoamide") OR ("glutamin") OR ("glutamine") OR ("glutamine residue") OR ("kabi glutamine") OR ("l glutamin") OR ("l glutamine") OR ("levo glutamin") OR ("levo glutamine") OR ("levoglutamide") OR ("levoglutamine") OR ("L Glutamine") OR ("nutrestore") OR ("xyndari") ) | 56966 |  |
| 51 | #28 AND #32 | 128554 |  |
| 54 | #39 OR #41 | 149128 |  |
| 56 | #51 OR #54 | 225783 |  |
| 58 | #56 AND #47 | 189 |  |
| CNKI | （篇关摘：肝切除 + 肝移植 + 手术（模糊））AND （篇关摘：谷氨酰胺 + L - 谷氨酰胺 + 麸氨酰胺（模糊）） | 366 |  |
| CQVIP | 题名或关键词=肝切除+肝移植+肝AND 题目或关键词=谷氨酰胺 | 133 |  |
| Wanfang | 题名或关键词:(肝切除 OR 肝移植 OR 手术) and 题名或关键词:(肝) and 题名或关键词:(谷氨酰胺) | 43 |  |
| Sinomed | ( "肝切除"[常用字段:智能] OR "肝移植"[常用字段:智能] OR "手术"[常用字段:智能]) AND "肝"[常用字段:智能] AND "谷氨酰胺"[常用字段:智能] | 78 |  |
