# Supplementary material for: Efficacy of glutamine supplementation in postoperative patients undergoing hepatic surgery: a systematic review and meta-analysis of randomized controlled trials
Source: Clinics (Sao Paulo). 2026 Sep 1;81:101086. doi: 10.1016/j.clinsp.2026.101086 (PMC13570316; doi:10.1016/j.clinsp.2026.101086)
Supplement: Supplementary file 2 [file mmc10.docx]

**CLINICS-D-25-01870_Supplementary Material**

**Supplement documents**

**Appendix 1** Search history.

| **Database** | **Search History** | **Search Results** | **Search Time** |
| --- | --- | --- | --- |
| PubMed |  |  |  |
| 1 | liver[Title/Abstract] OR hepatic[Title/Abstract] | 1,117,617 | 2025/7/24 |
| 2 | "Ghost Surgery" OR "operation" OR "operation care" OR "operative intervention" OR "Operative Procedure" OR "operative repair" OR "operative restoration" OR "operative surgery" OR "operative surgical procedure" OR "operative surgical procedures" OR "operative treatment" OR "research surgery" OR "resection" OR "surgery" OR "surgical care" OR "surgical correction" OR "surgical diagnosis" OR "surgical diagnostic techniques" OR "surgical exposure" OR "surgical intervention" OR "surgical management" OR "surgical operation" OR "surgical practice" OR "Surgical Procedure" OR "surgical repair" OR "surgical research" OR "surgical restoration" OR "surgical service" OR "surgical speciality" OR "surgical specialties" OR "surgical specialty" OR "surgical therapy" OR "surgical treatment" OR "preoperative" OR "postoperative" OR "perioperative" | 2,611,795 | 2025/7/24 |
| 3 | liver[MeSH Terms] | 479678 | 2025/7/24 |
| 5 | surgery[MeSH Terms] OR operation[MeSH Terms] | 3649031 | 2025/7/24 |
| 6 | hepatectomy[MeSH Terms] | 35203 | 2025/7/24 |
| 7 | "hemihepatectomy"[Title/Abstract] OR "hepatic resection"[Title/Abstract] OR "hepatic surgery"[Title/Abstract] OR "liver operation"[Title/Abstract] OR "liver resection"[Title/Abstract] OR "liver surgery"[Title/Abstract] | 23544 | 2025/7/24 |
| 8 | Liver Transplantation[MeSH Terms] | 65881 | 2025/7/24 |
| 9 | "Auxiliary Liver Transplantation"[Title/Abstract] OR "Hepatic Graft"[Title/Abstract] OR "Hepatic Transplant"[Title/Abstract] OR "Hepatic Transplantation"[Title/Abstract] OR "Liver Graft"[Title/Abstract] OR "Liver Grafting"[Title/Abstract] OR "Liver heterotopic Transplantation"[Title/Abstract] OR "Liver Orthotopic Graft"[Title/Abstract] OR "Liver Orthotopic Transplantation"[Title/Abstract] OR "Liver Tissue Transplantation"[Title/Abstract] OR "Liver Transplant"[Title/Abstract] OR "Liver Transplantation"[Title/Abstract] OR "Orthotopic Liver Transplantation"[Title/Abstract] | 74203 | 2025/7/24 |
| 10 | glutamine[MeSH Terms] | 20143 | 2025/7/24 |
| 11 | "acutil"[Title/Abstract] OR "adamin G"[Title/Abstract] OR "dl glutamine"[Title/Abstract] OR "D Glutamine"[Title/Abstract] OR "endari"[Title/Abstract] OR "glumin"[Title/Abstract] OR "glutamic acid 5 amide"[Title/Abstract] OR "glutamic acid monoamide"[Title/Abstract] OR "glutamin"[Title/Abstract] OR "glutamine"[Title/Abstract] OR "glutamine residue"[Title/Abstract] OR "kabi glutamine"[Title/Abstract] OR "l glutamin"[Title/Abstract] OR "l glutamine"[Title/Abstract] OR "levo glutamin"[Title/Abstract] OR "levo glutamine"[Title/Abstract] OR "levoglutamide"[Title/Abstract] OR "levoglutamine"[Title/Abstract] OR "L Glutamine"[Title/Abstract] OR "nutrestore"[Title/Abstract] OR "xyndari"[Title/Abstract] | 44127 | 2025/7/24 |
| 12 | (((#1 OR #3) AND (#2 OR #5)) OR (#6 OR #7) OR (#8 OR #9)) AND (#10 OR #11) | 644 | 2025/7/24 |
| Embase | | | |
| 1 | 'liver transplantation'/exp | 146739 | 2025/7/24 |
| 2 | 'auxiliary liver transplantation':ti,ab,kw OR 'hepatic graft':ti,ab,kw OR 'hepatic transplant':ti,ab,kw OR 'hepatic transplantation':ti,ab,kw OR 'liver graft':ti,ab,kw OR 'liver grafting':ti,ab,kw OR 'liver heterotopic transplantation':ti,ab,kw OR 'liver orthotopic graft':ti,ab,kw OR 'liver orthotopic transplantation':ti,ab,kw OR 'liver tissue transplantation':ti,ab,kw OR 'liver transplant':ti,ab,kw OR 'liver transplantation':ti,ab,kw OR 'orthotopic liver transplantation':ti,ab,kw | 126648 | 2025/7/24 |
| 3 | 'hepatectomy'/exp | 74069 | 2025/7/24 |
| 4 | 'hemihepatectomy':ti,ab,kw OR 'hepatic resection':ti,ab,kw OR 'hepatic surgery':ti,ab,kw OR 'liver operation':ti,ab,kw OR 'liver resection':ti,ab,kw OR 'liver surgery':ti,ab,kw | 36250 | 2025/7/24 |
| 5 | 'surgery'/exp OR 'operation'/exp | 6394650 | 2025/7/24 |
| 6 | 'ghost surgery':ti,ab,kw OR 'operation':ti,ab,kw OR 'operation care':ti,ab,kw OR 'operative intervention':ti,ab,kw OR 'operative procedure':ti,ab,kw OR 'operative repair':ti,ab,kw OR 'operative restoration':ti,ab,kw OR 'operative surgery':ti,ab,kw OR 'operative surgical procedure':ti,ab,kw OR 'operative surgical procedures':ti,ab,kw OR 'operative treatment':ti,ab,kw OR 'research surgery':ti,ab,kw OR 'resection':ti,ab,kw OR 'surgery':ti,ab,kw OR 'surgical care':ti,ab,kw OR 'surgical correction':ti,ab,kw OR 'surgical diagnosis':ti,ab,kw OR 'surgical diagnostic techniques':ti,ab,kw OR 'surgical exposure':ti,ab,kw OR 'surgical intervention':ti,ab,kw OR 'surgical management':ti,ab,kw OR 'surgical operation':ti,ab,kw OR 'surgical practice':ti,ab,kw OR 'surgical procedure':ti,ab,kw OR 'surgical repair':ti,ab,kw OR 'surgical research':ti,ab,kw OR 'surgical restoration':ti,ab,kw OR 'surgical service':ti,ab,kw OR 'surgical speciality':ti,ab,kw OR 'surgical specialties':ti,ab,kw OR 'surgical specialty':ti,ab,kw OR 'surgical therapy':ti,ab,kw OR 'surgical treatment':ti,ab,kw OR 'preoperative':ti,ab,kw OR 'postoperative':ti,ab,kw OR 'perioperative':ti,ab,kw | 3549985 | 2025/7/24 |
| 7 | 'glutamine'/exp | 54285 | 2025/7/24 |
| 8 | 'acutil':ti,ab,kw OR 'adamin g':ti,ab,kw OR 'dl glutamine':ti,ab,kw OR 'd glutamine':ti,ab,kw OR 'endari':ti,ab,kw OR 'glumin':ti,ab,kw OR 'glutamic acid 5 amide':ti,ab,kw OR 'glutamic acid monoamide':ti,ab,kw OR 'glutamin':ti,ab,kw OR 'glutamine':ti,ab,kw OR 'glutamine residue':ti,ab,kw OR 'kabi glutamine':ti,ab,kw OR 'l glutamin':ti,ab,kw OR 'levo glutamin':ti,ab,kw OR 'levo glutamine':ti,ab,kw OR 'levoglutamide':ti,ab,kw OR 'levoglutamine':ti,ab,kw OR 'l glutamine':ti,ab,kw OR 'nutrestore':ti,ab,kw OR 'xyndari':ti,ab,kw | 53395 | 2025/7/24 |
| 9 | 'liver'/exp | 742998 | 2025/7/24 |
| 10 | liver:ti,ab,kw OR hepatic:ti,ab,kw | 1539618 | 2025/7/24 |
| 11 | ((#9 OR #10) AND (#5 OR #6) OR #3 OR #4 OR #1 OR #2) AND (#7 OR #8) | 1093 | 2025/7/24 |
| Cochrane |  |  |  |
| 1 | MeSH descriptor: [Liver] explode all trees | 4633 |  |
| 2 | (liver OR hepatic):ti,ab,kw | 78481 |  |
| 3 | MeSH descriptor: [General Surgery] explode all trees | 513 |  |
| 4 | ('Ghost Surgery' OR 'operation' OR 'operation care' OR 'operative intervention' OR 'Operative Procedure' OR 'operative repair' OR 'operative restoration' OR 'operative surgery' OR 'operative surgical procedure' OR 'operative surgical procedures' OR 'operative treatment' OR 'research surgery' OR 'resection' OR 'surgery' OR 'surgical care' OR 'surgical correction' OR 'surgical diagnosis' OR 'surgical diagnostic techniques' OR 'surgical exposure' OR 'surgical intervention' OR 'surgical management' OR 'surgical operation' OR 'surgical practice' OR 'Surgical Procedure' OR 'surgical repair' OR 'surgical research' OR 'surgical restoration' OR 'surgical service' OR 'surgical speciality' OR 'surgical specialties' OR 'surgical specialty' OR 'surgical therapy' OR 'surgical treatment' OR 'preoperative' OR 'postoperative' OR 'perioperative'):ti,ab,kw | 402095 |  |
| 5 | MeSH descriptor: [Hepatectomy] explode all trees | 971 |  |
| 6 | ('hemihepatectomy' OR 'hepatic resection' OR 'hepatic surgery' OR 'liver operation' OR 'liver resection' OR 'liver surgery'):ti,ab,kw | 14779 |  |
| 7 | MeSH descriptor: [Liver Transplantation] explode all trees | 1708 |  |
| 8 | ('Auxiliary Liver Transplantation' OR 'Hepatic Graft' OR 'Hepatic Transplant' OR 'Hepatic Transplantation' OR 'Liver Graft' OR 'Liver Grafting' OR 'Liver heterotopic Transplantation' OR 'Liver Orthotopic Graft' OR 'Liver Orthotopic Transplantation' OR 'Liver Tissue Transplantation' OR 'Liver Transplant' OR 'Liver Transplantation' OR 'Orthotopic Liver Transplantation'):ti,ab,kw | 8232 |  |
| 9 | MeSH descriptor: [Glutamine] explode all trees | 828 |  |
| 10 | ('acutil' OR 'adamin G' OR 'dl glutamine' OR 'D Glutamine' OR 'endari' OR 'glumin' OR 'glutamic acid 5 amide' OR 'glutamic acid monoamide' OR 'glutamin' OR 'glutamine' OR 'glutamine residue' OR 'kabi glutamine' OR 'l glutamin' OR 'l glutamine' OR 'levo glutamin' OR 'levo glutamine' OR 'levoglutamide' OR 'levoglutamine' OR 'L Glutamine' OR 'nutrestore' OR 'xyndari'):ti,ab,kw | 2419 |  |
| 11 | ((#1 OR #2) AND (#3 OR #4) OR #5 OR #6 OR #7 OR #8) AND (#9 OR #10) | 52 |  |
| WOS |  |  |  |
| 28 | TS=((liver OR hepatic) ) | 1247718 |  |
| 32 | TS=(("Ghost Surgery") OR ("operation") OR ("operation care") OR ("operative intervention") OR ("Operative Procedure") OR ("operative repair") OR ("operative restoration") OR ("operative surgery") OR ("operative surgical procedure") OR ("operative surgical procedures") OR ("operative treatment") OR ("research surgery") OR ("resection") OR ("surgery") OR ("surgical care") OR ("surgical correction") OR ("surgical diagnosis") OR ("surgical diagnostic techniques") OR ("surgical exposure") OR ("surgical intervention") OR ("surgical management") OR ("surgical operation") OR ("surgical practice") OR ("Surgical Procedure") OR ("surgical repair") OR ("surgical research") OR ("surgical restoration") OR ("surgical service") OR ("surgical speciality") OR ("surgical specialties") OR ("surgical specialty") OR ("surgical therapy") OR ("surgical treatment") OR ("preoperative") OR ("postoperative") OR ("perioperative”)) | 3534317 |  |
| 39 | TS=(("Auxiliary Liver Transplantation") OR ("Hepatic Graft") OR ("Hepatic Transplant") OR ("Hepatic Transplantation") OR ("Liver Graft") OR ("Liver Grafting") OR ("Liver heterotopic Transplantation") OR ("Liver Orthotopic Graft") OR ("Liver Orthotopic Transplantation") OR ("Liver Tissue Transplantation") OR ("Liver Transplant") OR ("Liver Transplantation") OR ("Orthotopic Liver Transplantation") ) | 122535 |  |
| 41 | TS=(("hemihepatectomy") OR ("hepatic resection") OR ("hepatic surgery") OR ("liver operation") OR ("liver resection") OR ("liver surgery")) | 30384 |  |
| 47 | TS=(("acutil") OR ("adamin G") OR ("dl glutamine") OR ("D Glutamine") OR ("endari") OR ("glumin") OR ("glutamic acid 5 amide") OR ("glutamic acid monoamide") OR ("glutamin") OR ("glutamine") OR ("glutamine residue") OR ("kabi glutamine") OR ("l glutamin") OR ("l glutamine") OR ("levo glutamin") OR ("levo glutamine") OR ("levoglutamide") OR ("levoglutamine") OR ("L Glutamine") OR ("nutrestore") OR ("xyndari") ) | 56966 |  |
| 51 | #28 AND #32 | 128554 |  |
| 54 | #39 OR #41 | 149128 |  |
| 56 | #51 OR #54 | 225783 |  |
| 58 | #56 AND #47 | 189 |  |
| CNKI | （篇关摘：肝切除 + 肝移植 + 手术（模糊））AND （篇关摘：谷氨酰胺 + L - 谷氨酰胺 + 麸氨酰胺（模糊）） | 366 |  |
| CQVIP | 题名或关键词=肝切除+肝移植+肝AND 题目或关键词=谷氨酰胺 | 133 |  |
| Wanfang | 题名或关键词:(肝切除 OR 肝移植 OR 手术) and 题名或关键词:(肝) and 题名或关键词:(谷氨酰胺) | 43 |  |
| Sinomed | ( "肝切除"[常用字段:智能] OR "肝移植"[常用字段:智能] OR "手术"[常用字段:智能]) AND "肝"[常用字段:智能] AND "谷氨酰胺"[常用字段:智能] | 78 |  |

**Appendix 2** Risk of bias in included studies.

**Appendix 3** Literature screening flowchart.

**Appendix 4** Supplementary form for basic information

| Author | Year | Surgical Procedure | Intervention | Outcome | Glutamine dose, timing, and formulation, |
| --- | --- | --- | --- | --- | --- |
| Yao et al.[5] | 2021 | LR | Nutritional Support + Ala-Gln | IgA, IgG, IgM, CD3, CD4, CD8, CD4/CD8, TNF-α, IL-1, IL-6, IL-8, | 20 g by intravenous drip, once a day, for 7 days |
|  |  |  | Nutritional Support |  |  |
| Tong et al.[19] | 2021 | LTx | Metronidazole + Gln | CRP, TNF-α, IL-6 | 20g by intravenous drip, once a day, for 14 days |
|  |  |  | Metronidazole |  |  |
| Wang et al.[20] | 2018 | LR | Gln | CD3, CD4, ALT, AST, TNF-α, IL-1 | 0.4 g/ (kg·d), intravenous infusion, for 7 days |
|  |  |  | Normal Saline |  |  |
| Gao et al.[21] | 2018 | LR | Enteral Nutrition + Ala-Gln | ALB, PAB, ALT, AST, CHE, AE | 20 g/d, intravenous, for 6 to 8 days |
|  |  |  | Enteral Nutrition |  |  |
| Li et al.[22] | 2019 | LR | PN + Ala-Gln | ALB, PAB, TP, ALT, AST, TBIL, CHE, CRP, TNF-α, IL-6, IL-8, AE | 10g/50ml,intravenous, for 7 days |
|  |  |  | PN |  |  |
| Wan et al.[23] | 2008 | LTx | TPN+Gln | IgA, IgG, IgM, CD3, CD8, CD4/CD8, AE | Glutamine accounts for 20% of parenteral nutrition and is administered intravenously from the second day after the operation for 7 days. |
|  |  |  | TPN |  |  |
| Zhu et al.[24] | 2020 | LR | Intravenous Nutrition + Ala-Gln | IgA, IgG, IgM, CD3, CD4/CD8 | 20g/d, intravenous, for 7 days |
|  |  |  | Intravenous Nutrition |  |  |
| Tian et al.[25] | 2012 | LR | PN + Ala-Gln | IgA, IgG, IgM, AE | Starting from the first day after the operation, intravenous drip was administered for 8 days. |
|  |  |  | PN |  |  |
| Wu et al.[26] | 2011 | LR | PN + Gln | ALB, ALT, AST, TBIL, AE | Starting from the third day after the operation, take orally, three times a day, 10 g each time, for 7 consecutive days |
|  |  |  | Intravenous Nutrition + Ala-Gln |  |  |
| Qin et al.[27] | 2011 | LR | Intravenous Nutrition | CD3, CD4, CD8 | 0.4 g/ (kg·d), intravenous infusion, for 7 consecutive days |
|  |  |  | PN |  |  |
| Wang et al.[28] | 2006 | LTx | TPN+Ala-Gln | CHE, TBIL, | Intravenous, branched-chain amino acids and Ala-Gln (20% Lipeptide supply), lipeptide provides approximately 35% of the total nitrogen for 7 consecutive days |
|  |  |  | TPN |  |  |
|  |  |  | TPN |  |  |
| Wang et al.[30] | 2006 | LTx | TPN+Ala-Gln | CD3, CD4/CD8 | Intravenous, Lipei provides approximately 35% of the total nitrogen. Non-protein calories: nitrogen =653 kJ: 1g, continuous for 7 days, intravenous |
|  |  |  | TPN |  |  |
| Lin et al.[31] | 2017 | LR | Gln | TNF-α, | 0.75 g/kg, intraoperative, injected via femoral vein |
|  |  |  | Sodium Chloride |  |  |
| Wang et al.[14] | 2020 | LR | PN + L-Alanylgutamine | IgA, IgG, IgM | 20g/d, intravenous, for 7 consecutive days |
|  |  |  | PN |  |  |
| Jiang et al.[32] | 2007 | LTx | Enteral Nutrition + Gln Dipeptide | AE | 0.3g/ (kg/d), enteral nutrition infusion through nasojejunal nutrition tube for 7 consecutive days |
|  |  |  | Enteral Nutrition |  |  |
| Barros et al.[33] | 2015 | LTx | L-Alanylgutamine | ALT, AST, TBIL | 50g, intravenous |
|  |  |  | Normal Saline |  |  |
| Qiu et al.[34] | 2009 | LTx | TPN+Gln | IgA, IgG, IgM, CD4, ALB, PAB, TP, ALT, AST, DBIL, AE | Starting from the second day after the operation, intravenous drip was administered for 7 days. |
|  |  |  | TPN |  |  |
| Richard et al.[15] | 2014 | LTx | EN+Gln | ALB, ALT, AST, TBIL, DBIL, CRP | Take orally 3 days before the operation and 4 days after the operation, 30 g/day |
|  |  |  | EN |  |  |

**Appendix 5** Forest plot of the meta-analysis on the impact of glutamine supplementation on postoperative mortality in postoperative liver surgery patients.

**Appendix 6** Subgroup analysis. Forest plot of the subgroup analysis on the impact of glutamine supplementation on immunoglobulin levels in postoperative liver surgery patients. Note: Pooled Standardized Mean Difference (SMD) with its 95% Confidence Interval (95% CI).

**Appendix 7** Sensitivity analysis. Forest plot of the sensitivity analysis on the impact of glutamine supplementation on *t*-cell subtypes in postoperative liver surgery patients.

**Appendix 8** Heterogeneity test.

Total Bilirubin (TB) ‒ Treatment Duration. Note: Pooled Standardized Mean Difference (SMD) with its 95% Confidence Interval (95% CI).

Total Bilirubin (TB) ‒ Detection Time. Note: Pooled Standardized Mean Difference (SMD) with its 95% Confidence Interval (95% CI).

Total Bilirubin (TB) ‒ Drug Formulation Subgroup. Note: Pooled Standardized Mean Difference (SMD) with its 95% Confidence Interval (95% CI).

Total Bilirubin (TB) ‒ Dosage Subgroup. Note: Pooled Standardized Mean Difference (SMD) with its 95% Confidence Interval (95% CI).

Prealbumin (PAB) - Funnel Plot.

Cholinesterase (CHE) ‒ Treatment Duration Subgroup. Note: Pooled Standardized Mean Difference (SMD) with its 95% Confidence Interval (95% CI).

Tumor Necrosis Factor-alpha (TNF-α) ‒ Treatment Duration Subgroup. Note: Pooled Standardized Mean Difference (SMD) with its 95% Confidence Interval (95% CI).

Tumor Necrosis Factor-alpha (TNF-α) ‒ Funnel Plot.

Total Bilirubin (TB) ‒ Funnel Plot.

Interleukin-6 (IL-6) ‒ Funnel Plot.

Interleukin-6 (IL-6) ‒ Detection Time Subgroup. Note: Pooled Standardized Mean Difference (SMD) with its 95% Confidence Interval (95% CI).

Interleukin-6 (IL-6) ‒ Dosage Subgroup. Note: Pooled Standardized Mean Difference (SMD) with its 95% Confidence Interval (95% CI).

Immunoglobulin M (IgM) ‒ Funnel Plot.

Immunoglobulin G (IgG) ‒ Treatment Duration Subgroup. Note: Pooled Standardized Mean Difference (SMD) with its 95% Confidence Interval (95% CI).

Immunoglobulin G (IgG) - Funnel Plot.

Immunoglobulin G (IgG) ‒ Detection Time Subgroup. Note: Pooled Standardized Mean Difference (SMD) with its 95% Confidence Interval (95% CI).

Immunoglobulin G (IgG) ‒ Dosage Subgroup. Note: Pooled Standardized Mean Difference (SMD) with its 95% Confidence Interval (95% CI).

Immunoglobulin A (IgA) ‒ Treatment Duration Subgroup. Note: Pooled Standardized Mean Difference (SMD) with its 95% Confidence Interval (95% CI).

Immunoglobulin A (IgA) ‒ Funnel Plot.

Immunoglobulin A (IgA) ‒ Detection Time Subgroup. Note: Pooled Standardized Mean Difference (SMD) with its 95% Confidence Interval (95% CI).

Immunoglobulin A (IgA) ‒ Dosage Subgroup. Note: Pooled standardized mean difference (SMD) with its 95% confidence interval (CI).

C-Reactive Protein (CRP) ‒ Funnel Plot.

C-Reactive Protein (CRP) ‒ Drug Formulation Subgroup. Note: Pooled Standardized Mean Difference (SMD) with its 95% Confidence Interval (95% CI).

Cholinesterase (CHE) ‒ Funnel Plot.

CD8+ T Cells ‒ Funnel Plot.

CD8+ T Cells ‒ Dosage Subgroup. Note: Pooled Standardized Mean Difference (SMD) with its 95% Confidence Interval (95% CI).

CD4+ T Cells ‒ Funnel Plot.

CD4+ T Cells - Detection Time Subgroup. Note: Pooled Standardized Mean Difference (SMD) with its 95% Confidence Interval (95% CI).

CD4+ T Cells ‒ Dosage Subgroup. Note: Pooled Standardized Mean Difference (SMD) with its 95% Confidence Interval (95% CI).

CD4/CD8 Ratio - Subgroup Analysis

Note: Pooled standardized mean difference (SMD) with its 95% confidence interval (CI).

CD4/CD8 Ratio - Funnel Plot

CD4/CD8 Ratio - Dosage Subgroup

Note: Pooled standardized mean difference (SMD) with its 95% confidence interval (CI).

CD3+ T Cells - Funnel Plot

CD3+ T Cells - Detection Time Subgroup

Note: Pooled standardized mean difference (SMD) with its 95% confidence interval (CI).

CD3+ T Cells - Dosage Subgroup

Note: Pooled standardized mean difference (SMD) with its 95% confidence interval (CI).

Aspartate Aminotransferase (AST) - Treatment Duration Subgroup

Note: Pooled standardized mean difference (SMD) with its 95% confidence interval (CI).

Aspartate Aminotransferase (AST) - Funnel Plot

Aspartate Aminotransferase (AST) - Detection Time Subgroup

Note: Pooled standardized mean difference (SMD) with its 95% confidence interval (CI).

Aspartate Aminotransferase (AST) - Drug Formulation Subgroup

Note: Pooled standardized mean difference (SMD) with its 95% confidence interval (CI).

Aspartate Aminotransferase (AST) - Dosage Subgroup

Note: Pooled standardized mean difference (SMD) with its 95% confidence interval (CI).

Alanine Aminotransferase (ALT) - Treatment Duration Subgroup

Note: Pooled standardized mean difference (SMD) with its 95% confidence interval (CI).

Alanine Aminotransferase (ALT) - Funnel Plot

Alanine Aminotransferase (ALT) - Detection Time Subgroup

Note: Pooled standardized mean difference (SMD) with its 95% confidence interval (CI).

Alanine Aminotransferase (ALT) - Drug Formulation Subgroup

Note: Pooled standardized mean difference (SMD) with its 95% confidence interval (CI).

Alanine Aminotransferase (ALT) - Dosage Subgroup

Note: Pooled standardized mean difference (SMD) with its 95% confidence interval (CI).

Albumin (ALB) - Treatment Duration Subgroup

Note: Pooled standardized mean difference (SMD) with its 95% confidence interval (CI).

Albumin (ALB) - Funnel Plot

Albumin (ALB) - Detection Time Subgroup

Note: Pooled standardized mean difference (SMD) with its 95% confidence interval (CI).

Albumin (ALB) - Drug Formulation Subgroup

Note: Pooled standardized mean difference (SMD) with its 95% confidence interval (CI).

Albumin (ALB) - Dosage Subgroup

Note: Pooled standardized mean difference (SMD) with its 95% confidence interval (CI).

Adverse Events (AE) - Funnel Plot

**Appendix 9 Results of Egger's test for publication bias**

| Egger's test | | | | | | | | | |
| --- | --- | --- | --- | --- | --- | --- | --- | --- | --- |
| Egger's test | IgA | IgG | IgM | CD3 | CD4 | CD8 | CD4/CD8 | CPR | TNF-α |
| *P* | 0.951 | 0.724 | 0.919 | 0.363 | 0.906 | 0.551 | 0.772 | 0.539 | 0.066 |
| Egger's test | IL6 | ALT | AST | CHE | TBIL | ALB | PAB | AE |  |
| *P* | 0.543 | 0.842 | 0.711 | 0.486 | 0.499 | 0.803 | 0.273 | 0.091 |  |
