# Supplementary material for: Efficacy of glutamine supplementation in postoperative patients undergoing hepatic surgery: a systematic review and meta-analysis of randomized controlled trials
Source: Clinics (Sao Paulo). 2026 Sep 1;81:101086. doi: 10.1016/j.clinsp.2026.101086 (PMC13570316; doi:10.1016/j.clinsp.2026.101086)
Supplement: Supplementary file 5 [file mmc4.docx]

**Appendix 4** Supplementary form for basic information

| Author | Year | Surgical Procedure | Intervention | Outcome | Glutamine dose, timing, and formulation, |
| --- | --- | --- | --- | --- | --- |
| Yao et al.[5] | 2021 | LR | Nutritional Support + Ala-Gln | IgA, IgG, IgM, CD3, CD4, CD8, CD4/CD8, TNF-α, IL-1, IL-6, IL-8, | 20 g by intravenous drip, once a day, for 7 days |
|  |  |  | Nutritional Support |  |  |
| Tong et al.[19] | 2021 | LTx | Metronidazole + Gln | CRP, TNF-α, IL-6 | 20g by intravenous drip, once a day, for 14 days |
|  |  |  | Metronidazole |  |  |
| Wang et al.[20] | 2018 | LR | Gln | CD3, CD4, ALT, AST, TNF-α, IL-1 | 0.4 g/ (kg·d), intravenous infusion, for 7 days |
|  |  |  | Normal Saline |  |  |
| Gao et al.[21] | 2018 | LR | Enteral Nutrition + Ala-Gln | ALB, PAB, ALT, AST, CHE, AE | 20 g/d, intravenous, for 6 to 8 days |
|  |  |  | Enteral Nutrition |  |  |
| Li et al.[22] | 2019 | LR | PN + Ala-Gln | ALB, PAB, TP, ALT, AST, TBIL, CHE, CRP, TNF-α, IL-6, IL-8, AE | 10g/50ml,intravenous, for 7 days |
|  |  |  | PN |  |  |
| Wan et al.[23] | 2008 | LTx | TPN+Gln | IgA, IgG, IgM, CD3, CD8, CD4/CD8, AE | Glutamine accounts for 20% of parenteral nutrition and is administered intravenously from the second day after the operation for 7 days. |
|  |  |  | TPN |  |  |
| Zhu et al.[24] | 2020 | LR | Intravenous Nutrition + Ala-Gln | IgA, IgG, IgM, CD3, CD4/CD8 | 20g/d, intravenous, for 7 days |
|  |  |  | Intravenous Nutrition |  |  |
| Tian et al.[25] | 2012 | LR | PN + Ala-Gln | IgA, IgG, IgM, AE | Starting from the first day after the operation, intravenous drip was administered for 8 days. |
|  |  |  | PN |  |  |
| Wu et al.[26] | 2011 | LR | PN + Gln | ALB, ALT, AST, TBIL, AE | Starting from the third day after the operation, take orally, three times a day, 10 g each time, for 7 consecutive days |
|  |  |  | Intravenous Nutrition + Ala-Gln |  |  |
| Qin et al.[27] | 2011 | LR | Intravenous Nutrition | CD3, CD4, CD8 | 0.4 g/ (kg·d), intravenous infusion, for 7 consecutive days |
|  |  |  | PN |  |  |
| Wang et al.[28] | 2006 | LTx | TPN+Ala-Gln | CHE, TBIL, | Intravenous, branched-chain amino acids and Ala-Gln (20% Lipeptide supply), lipeptide provides approximately 35% of the total nitrogen for 7 consecutive days |
|  |  |  | TPN |  |  |
|  |  |  | TPN |  |  |
| Wang et al.[30] | 2006 | LTx | TPN+Ala-Gln | CD3, CD4/CD8 | Intravenous, Lipei provides approximately 35% of the total nitrogen. Non-protein calories: nitrogen =653 kJ: 1g, continuous for 7 days, intravenous |
|  |  |  | TPN |  |  |
| Lin et al.[31] | 2017 | LR | Gln | TNF-α, | 0.75 g/kg, intraoperative, injected via femoral vein |
|  |  |  | Sodium Chloride |  |  |
| Wang et al.[14] | 2020 | LR | PN + L-Alanylgutamine | IgA, IgG, IgM | 20g/d, intravenous, for 7 consecutive days |
|  |  |  | PN |  |  |
| Jiang et al.[32] | 2007 | LTx | Enteral Nutrition + Gln Dipeptide | AE | 0.3g/ (kg/d), enteral nutrition infusion through nasojejunal nutrition tube for 7 consecutive days |
|  |  |  | Enteral Nutrition |  |  |
| Barros et al.[33] | 2015 | LTx | L-Alanylgutamine | ALT, AST, TBIL | 50g, intravenous |
|  |  |  | Normal Saline |  |  |
| Qiu et al.[34] | 2009 | LTx | TPN+Gln | IgA, IgG, IgM, CD4, ALB, PAB, TP, ALT, AST, DBIL, AE | Starting from the second day after the operation, intravenous drip was administered for 7 days. |
|  |  |  | TPN |  |  |
| Richard et al.[15] | 2014 | LTx | EN+Gln | ALB, ALT, AST, TBIL, DBIL, CRP | Take orally 3 days before the operation and 4 days after the operation, 30 g/day |
|  |  |  | EN |  |  |
