# Supplementary material for: Efficacy of glutamine supplementation in postoperative patients undergoing hepatic surgery: a systematic review and meta-analysis of randomized controlled trials
Source: Clinics (Sao Paulo). 2026 Sep 1;81:101086. doi: 10.1016/j.clinsp.2026.101086 (PMC13570316; doi:10.1016/j.clinsp.2026.101086)
Supplement: Supplementary file 6 [file mmc6.docx]

**Appendix 6 Subgroup Analysis**

Forest Plot of the Subgroup Analysis on the Impact of Glutamine Supplementation on Immunoglobulin Levels in Postoperative Liver Surgery Patients

Note: Pooled standardized mean difference (SMD) with its 95% confidence interval (CI).

 Forest Plot of the Subgroup Analysis on the Impact of Glutamine Supplementation on T-Cell Subtypes in Postoperative Liver Surgery Patients

Note: Pooled standardized mean difference (SMD) with its 95% confidence interval (CI).

 Forest Plot of the Subgroup Analysis on the Impact of Glutamine Supplementation on Inflammatory Cytokines in Postoperative Liver Surgery Patients

Note: Pooled standardized mean difference (SMD) with its 95% confidence interval (CI).

 Forest Plot of the Subgroup Analysis on the Impact of Glutamine Supplementation on Liver Function in Postoperative Liver Surgery Patients

Note: Pooled standardized mean difference (SMD) with its 95% confidence interval (CI).

 Forest Plot of the Subgroup Analysis on the Impact of Glutamine Supplementation on Protein Levels in Postoperative Liver Surgery Patients

Note: Pooled standardized mean difference (SMD) with its 95% confidence interval (CI).

 Forest Plot of the Subgroup Analysis on the Impact of Glutamine Supplementation on AEs in Postoperative Liver Surgery Patients

Note: Pooled standardized mean difference (SMD) with its 95% confidence interval (CI).
