# Supplementary material for: Efficacy of glutamine supplementation in postoperative patients undergoing hepatic surgery: a systematic review and meta-analysis of randomized controlled trials
Source: Clinics (Sao Paulo). 2026 Sep 1;81:101086. doi: 10.1016/j.clinsp.2026.101086 (PMC13570316; doi:10.1016/j.clinsp.2026.101086)
Supplement: Supplementary file 7 [file mmc7.docx]

**Appendix 7 Sensitivity Analysis**

Forest Plot of the Sensitivity Analysis on the Impact of Glutamine Supplementation on Immunoglobulin Levels in Postoperative Liver Surgery Patients

Forest Plot of the Sensitivity Analysis on the Impact of Glutamine Supplementation on T-Cell Subtypes in Postoperative Liver Surgery Patients

Forest Plot of the Sensitivity Analysis on the Impact of Glutamine Supplementation on Inflammatory Cytokines in Postoperative Liver Surgery Patients

Forest Plot of the Sensitivity Analysis on the Impact of Glutamine Supplementation on Liver Function in Postoperative Liver Surgery Patients

 Forest Plot of the Sensitivity Analysis on the Impact of Glutamine Supplementation on Protein Levels in Postoperative Liver Surgery Patients

 Forest Plot of the Sensitivity Analysis on the Impact of Glutamine Supplementation on AEs in Postoperative Liver Surgery Patients
