# Supplementary material for: Efficacy of glutamine supplementation in postoperative patients undergoing hepatic surgery: a systematic review and meta-analysis of randomized controlled trials
Source: Clinics (Sao Paulo). 2026 Sep 1;81:101086. doi: 10.1016/j.clinsp.2026.101086 (PMC13570316; doi:10.1016/j.clinsp.2026.101086)
Supplement: Supplementary file 8 [file mmc8.docx]

**Appendix 8** Heterogeneity test

Total Bilirubin (TB) - Treatment Duration

Note: Pooled standardized mean difference (SMD) with its 95% confidence interval (CI).

Total Bilirubin (TB) - Detection Time

Note: Pooled standardized mean difference (SMD) with its 95% confidence interval (CI).

Total Bilirubin (TB) - Drug Formulation Subgroup

Note: Pooled standardized mean difference (SMD) with its 95% confidence interval (CI).

Total Bilirubin (TB) - Dosage Subgroup

Note: Pooled standardized mean difference (SMD) with its 95% confidence interval (CI).

Prealbumin (PAB) - Funnel Plot

Cholinesterase (CHE) - Treatment Duration Subgroup

Note: Pooled standardized mean difference (SMD) with its 95% confidence interval (CI).

Tumor Necrosis Factor-alpha (TNF-α) - Treatment Duration Subgroup

Note: Pooled standardized mean difference (SMD) with its 95% confidence interval (CI).

Tumor Necrosis Factor-alpha (TNF-α) - Funnel Plot

Total Bilirubin (TB) - Funnel Plot

Interleukin-6 (IL-6) - Funnel Plot

Interleukin-6 (IL-6) - Detection Time Subgroup

Note: Pooled standardized mean difference (SMD) with its 95% confidence interval (CI).

Interleukin-6 (IL-6) - Dosage Subgroup

Note: Pooled standardized mean difference (SMD) with its 95% confidence interval (CI).

Immunoglobulin M (IgM) - Funnel Plot

Immunoglobulin G (IgG) - Treatment Duration Subgroup

Note: Pooled standardized mean difference (SMD) with its 95% confidence interval (CI).

Immunoglobulin G (IgG) - Funnel Plot

Immunoglobulin G (IgG) - Detection Time Subgroup

Note: Pooled standardized mean difference (SMD) with its 95% confidence interval (CI).

Immunoglobulin G (IgG) - Dosage Subgroup

Note: Pooled standardized mean difference (SMD) with its 95% confidence interval (CI).

Immunoglobulin A (IgA) - Treatment Duration Subgroup

Note: Pooled standardized mean difference (SMD) with its 95% confidence interval (CI).

Immunoglobulin A (IgA) - Funnel Plot

Immunoglobulin A (IgA) - Detection Time Subgroup

Note: Pooled standardized mean difference (SMD) with its 95% confidence interval (CI).

Immunoglobulin A (IgA) - Dosage Subgroup

Note: Pooled standardized mean difference (SMD) with its 95% confidence interval (CI).

C-Reactive Protein (CRP) - Funnel Plot

C-Reactive Protein (CRP) - Drug Formulation Subgroup

Note: Pooled standardized mean difference (SMD) with its 95% confidence interval (CI).

Cholinesterase (CHE) - Funnel Plot

CD8+ T Cells - Funnel Plot

CD8+ T Cells - Dosage Subgroup

Note: Pooled standardized mean difference (SMD) with its 95% confidence interval (CI).

CD4+ T Cells - Funnel Plot

CD4+ T Cells - Detection Time Subgroup

Note: Pooled standardized mean difference (SMD) with its 95% confidence interval (CI).

CD4+ T Cells - Dosage Subgroup

Note: Pooled standardized mean difference (SMD) with its 95% confidence interval (CI).

CD4/CD8 Ratio - Subgroup Analysis

Note: Pooled standardized mean difference (SMD) with its 95% confidence interval (CI).

CD4/CD8 Ratio - Funnel Plot

CD4/CD8 Ratio - Dosage Subgroup

Note: Pooled standardized mean difference (SMD) with its 95% confidence interval (CI).

CD3+ T Cells - Funnel Plot

CD3+ T Cells - Detection Time Subgroup

Note: Pooled standardized mean difference (SMD) with its 95% confidence interval (CI).

CD3+ T Cells - Dosage Subgroup

Note: Pooled standardized mean difference (SMD) with its 95% confidence interval (CI).

Aspartate Aminotransferase (AST) - Treatment Duration Subgroup

Note: Pooled standardized mean difference (SMD) with its 95% confidence interval (CI).

Aspartate Aminotransferase (AST) - Funnel Plot

Aspartate Aminotransferase (AST) - Detection Time Subgroup

Note: Pooled standardized mean difference (SMD) with its 95% confidence interval (CI).

Aspartate Aminotransferase (AST) - Drug Formulation Subgroup

Note: Pooled standardized mean difference (SMD) with its 95% confidence interval (CI).

Aspartate Aminotransferase (AST) - Dosage Subgroup

Note: Pooled standardized mean difference (SMD) with its 95% confidence interval (CI).

Alanine Aminotransferase (ALT) - Treatment Duration Subgroup

Note: Pooled standardized mean difference (SMD) with its 95% confidence interval (CI).

Alanine Aminotransferase (ALT) - Funnel Plot

Alanine Aminotransferase (ALT) - Detection Time Subgroup

Note: Pooled standardized mean difference (SMD) with its 95% confidence interval (CI).

Alanine Aminotransferase (ALT) - Drug Formulation Subgroup

Note: Pooled standardized mean difference (SMD) with its 95% confidence interval (CI).

Alanine Aminotransferase (ALT) - Dosage Subgroup

Note: Pooled standardized mean difference (SMD) with its 95% confidence interval (CI).

Albumin (ALB) - Treatment Duration Subgroup

Note: Pooled standardized mean difference (SMD) with its 95% confidence interval (CI).

Albumin (ALB) - Funnel Plot

Albumin (ALB) - Detection Time Subgroup

Note: Pooled standardized mean difference (SMD) with its 95% confidence interval (CI).

Albumin (ALB) - Drug Formulation Subgroup

Note: Pooled standardized mean difference (SMD) with its 95% confidence interval (CI).

Albumin (ALB) - Dosage Subgroup

Note: Pooled standardized mean difference (SMD) with its 95% confidence interval (CI).

Adverse Events (AE) - Funnel Plot
