# Supplementary material for: Efficacy of glutamine supplementation in postoperative patients undergoing hepatic surgery: a systematic review and meta-analysis of randomized controlled trials
Source: Clinics (Sao Paulo). 2026 Sep 1;81:101086. doi: 10.1016/j.clinsp.2026.101086 (PMC13570316; doi:10.1016/j.clinsp.2026.101086)
Supplement: Supplementary file 9 [file mmc9.docx]

| Egger's test | | | | | | | | | |
| --- | --- | --- | --- | --- | --- | --- | --- | --- | --- |
| Egger's test | IgA | IgG | IgM | CD3 | CD4 | CD8 | CD4/CD8 | CPR | TNF-α |
| *P* | 0.951 | 0.724 | 0.919 | 0.363 | 0.906 | 0.551 | 0.772 | 0.539 | 0.066 |
| Egger's test | IL6 | ALT | AST | CHE | TBIL | ALB | PAB | AE |  |
| *P* | 0.543 | 0.842 | 0.711 | 0.486 | 0.499 | 0.803 | 0.273 | 0.091 |  |

**Appendix 9 Results of Egger's test for publication bias**
