# Supplementary material for: Efficacy of glutamine supplementation in postoperative patients undergoing hepatic surgery: a systematic review and meta-analysis of randomized controlled trials
Source: Clinics (Sao Paulo). 2026 Sep 1;81:101086. doi: 10.1016/j.clinsp.2026.101086 (PMC13570316; doi:10.1016/j.clinsp.2026.101086)
Supplement: Supplementary file 11 [file mmc3.docx]

**Appendix 3 Literature Screening Flowchart**
